# Spatiotemporal analysis of dystrophin expression during muscle repair

**DOI:** 10.1186/s13395-025-00398-y

**Published:** 2025-10-02

**Authors:** John C.W. Hildyard, Liberty E. Roskrow, Dominic J. Wells, Richard J. Piercy

**Affiliations:** 1 Comparative Neuromuscular Diseases Laboratory, Department of Clinical Science and Services, Royal Veterinary College, London, UK; 2 Department of Comparative Biomedical Sciences, Royal Veterinary College, London, UK

## Abstract

**Background:**

Dystrophin mRNA is produced from a very large genetic locus and transcription of a single mRNA requires approximately 16 h. This prolonged interval between initiation and completion results in unusual transcriptional behaviour: in skeletal muscle, myonuclei express dystrophin continuously and robustly, yet degrade mature transcripts shortly after completion. Consequently, most dystrophin mRNA is nascent, not mature. This implies expression is principally controlled post-transcriptionally, a mechanism that circumvents transcriptional delay, allowing rapid responses to change in demand. Dystrophin protein is however highly stable, with slow turnover: in healthy muscle, despite constant production of dystrophin mRNA, demand is low and the need for responsive expression is minimal. We reasoned this system instead exists to control dystrophin expression during rare periods of elevated but changing demand, such as during muscle development or repair, when newly formed fibres must establish sarcolemmal dystrophin rapidly.

**Methods:**

We assessed dystrophin mRNA (both nascent and mature) and dystrophin protein in regenerating skeletal muscle following injury, using a combination of qPCR, immunofluorescence and in-situ hybridisation to determine timing and location of expression during the repair process.

**Results:**

We reveal a complex program that suggests control at multiple levels: nascent transcription is detectable even prior to overt myoblast fusion, suggesting cells ‘pay in advance’ to minimise subsequent delay. During myotube differentiation and maturation, when sarcolemmal demands are high, initiation increases only modestly while mature transcript stability increases markedly to generate high numbers of mature dystrophin transcripts, a state that persists until repair is complete, when oversupply and degradation resumes.

**Conclusion:**

Our data demonstrate that dystrophin mRNA is indeed chiefly controlled by turnover, not initiation: degradation consequently represents a potential therapeutic target for maximising efficacy of even modest dystrophin restoration.

**Supplementary Information:**

The online version contains supplementary material available at 10.1186/s13395-025-00398-y.

## Introduction

**Fig. 1:**
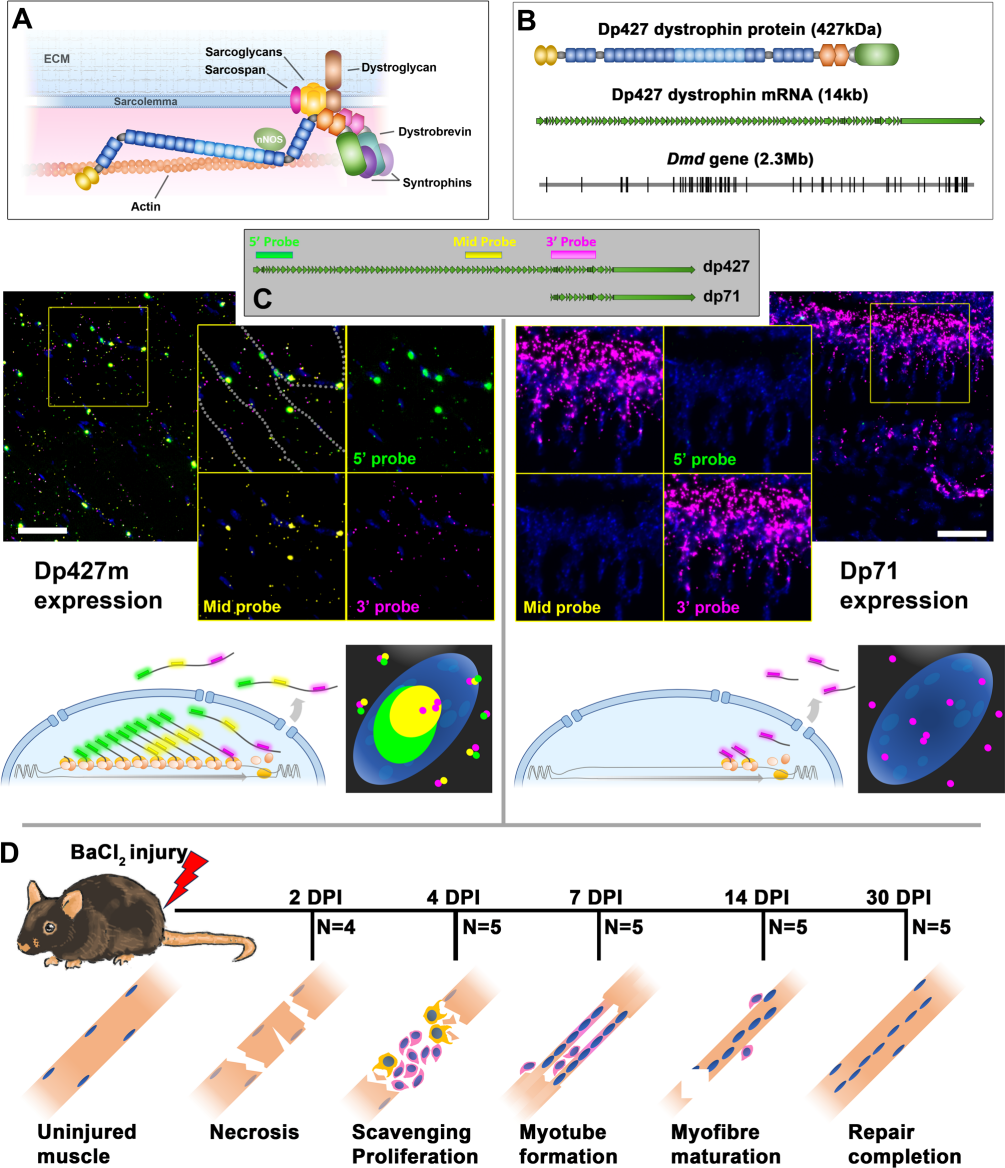
Overview. (**A**) Dystrophin (dp427) protein localises to the sarcolemma as a core component of the dystrophin-associated glycoprotein complex (DAGC), physically connecting cytoskeletal actin to sarcolemmal-spanning dystroglycan, and localising multiple additional proteins. (**B**) Sizes/lengths of dystrophin sequences: dp427 protein (~ 1300 amino acids, 427 kDa) and mRNA (14 kb) are very large, but the *Dmd* gene (2.3 Mb) is enormous. (**C**) Use of multiplex RNAscope FISH probes to dystrophin 5’, middle and 3’ regions distinguishes muscle dp427 (all three probes) from dp71 (3’ probe only). Use of these probes in muscle (left hand panels, dotted lines: myofibre boundaries) reveals a unique dp427 signature: myonuclei exhibit strong nuclear 5’ and middle probe labelling, but only punctate 3’ probe, while sarcoplasm hosts small puncta of all three probes. This is consistent with large numbers of nascent transcripts within myonuclei, but more modest mature transcript numbers (see cartoon). By comparison, cells expressing dp71 (such as neuroblasts within the developing brain -right panels) label with 3’ probe only, and do not show prominent nuclear foci. Scalebars: 50 μm. Experimental model (**D**): WT mice were injured via BaCl_2_ injection (left tibialis anterior), and muscles (injured and uninjured) were collected at the indicated days post injury (DPI), consistent with necrosis (day 2), proliferation/scavenging (day 4), early regeneration (day 7), late regeneration (day 14) and repair completion (day 30). Animal numbers for each time-point are indicated

Dystrophin is essential for muscle health: in skeletal muscle myofibres this 427 kDa protein (dp427) is found at the sarcolemma as a core component of the dystrophin associated glycoprotein complex (DAGC), a macromolecular assembly that links cytoskeletal actin and microtubule networks to the extracellular matrix (ECM). The complex forms a physical bridge between the myofibre cytoskeleton and the ECM environment, distributing contractile force and buffering the sarcolemmal membrane against the stress of muscle activity (Fig. 1A). Without dp427, myofibres are vulnerable to contraction-induced damage, and loss or insufficiency of this protein results in the severe muscle wasting condition Duchenne muscular dystrophy (DMD), a fatal, incurable disease characterised by repeated cycles of muscle damage, regeneration and concomitant inflammation, fibrosis and atrophy.

Dystrophin is transcribed from an exceptionally long gene (*Dmd*): at ~ 2.3 megabases, it is one of the longest genes in the mammalian genome (Fig. 1B), a feature that presents significant biological challenges. Much of the gene is intronic (several introns are > 100 kb) and the 14 kb mature mRNA represents less than 0.5% of total *Dmd* sequence, but production of a single dp427 transcript still requires all 2.3 Mb to be transcribed in full. Early work by Tennyson et al. demonstrated that this takes approximately 16 hours [1], implying a mean transcription rate of 40 bases.sec^− 1^ (agreeing with estimates for other long genes [2, 3]). This lengthy transcription time therefore likely represents essentially uninterrupted polymerase activity (later work by Gherardi et al. [4] confirmed that polymerase pausing was not a significant contributor to this long transcription time). Tennyson et al. further showed that splicing of dp427 occurs co-transcriptionally, with spliced 5’ sequence detectable significantly before any 3’ sequence [1], and work by Gazzoli et al. demonstrated this splicing is complex [5], with specific groups of exons spliced before others (some long introns are moreover spliced in sequential, piecemeal fashion). Notably, however, the dp427 transcript is not alternatively spliced [6]. A remarkable conclusion of these studies is that 5’ sequence is not simply transcribed several hours before 3’ sequence, but also remains in excess of 3’ sequence persistently: in mature, healthy skeletal muscle where dystrophin expression should be essentially at steady-state, spliced dystrophin 5’ sequence (exons 1–2) is detectable at markedly higher levels than 3’ sequence (exons 62–63) [7, 8]. This phenomenon has been observed with dystrophin in humans, mice and dogs, and has been termed ‘transcript imbalance’ [9]. Imbalance is typically more dramatic in dystrophic muscle, leading to further suggestions that it might be pathologically relevant. An alternative hypothesis is that this reflects inherent instability of mature dp427 mRNAs, with a half-life substantially shorter than the time required for production. Under such a scenario, initiated but not-yet-complete dp427 transcripts (bearing spliced 5’ sequence but not 3’) might be present in considerable numbers, while mature transcripts (bearing both spliced 5’ and 3’ sequence) would be in the minority. In essence, even under steady-state healthy conditions, most dp427 mRNA would be nascent, not mature.

We have shown this is indeed the case, using a multiplex single-molecule fluorescence in situ hybridisation (smFISH) approach to label different regions (5’, central and 3’) of the long dp427 mRNA (Fig. 1C: this also allows shorter isoforms such as dp71 to be distinguished -righthand panels) [8, 10]. In healthy skeletal muscle fibres, individual mature sarcoplasmic mRNAs are present near the sarcolemma in modest numbers, labelled in a punctate manner with all three probes. Myonuclei, in contrast, are host to large, intense foci of dystrophin 5’ probe, but only scattered punctate foci of 3’ probe, while signal from probes to the central dp427 sequence falls between these two extremes (Fig. 1C, lefthand panels). This labelling pattern is consistent with high numbers of immature mRNAs (~ 20–40 per nucleus [8]) at various stages of transcription: ‘transcript imbalance’ is an inevitable consequence of ongoing transcriptional initiation, long transcription time, and short mature mRNA half-life (as discussed elsewhere [11]).

This model is puzzling, implying nuclei continuously commit to the 16 h (and 2.3 million base incorporation events) required for each transcript, only to degrade those transcripts shortly after completion. The turnover of dystrophin protein in skeletal muscle is also slow (weeks to months [12, 13]), thus most transcripts might be wholly surplus to requirements. Supply of dystrophin mRNA substantially outstrips demand, and in healthy muscle at steady state, it seems plausible that the bulk of dp427 mRNA produced is not meaningfully translated at all (translational inactivity could itself potentiate transcript degradation [14, 15]).

We proposed [11] that this system allows fine tuning of dystrophin transcripts over short timescales: instead of initiating transcription in response to demand (necessarily incurring a 16 h delay), myonuclei might express dystrophin constitutively regardless of demand, and adjust mRNA levels post-transcriptionally via control of degradation. By sacrificing efficiency for control, increases in demand could be met rapidly by stabilizing recently completed transcripts initiated 16 h previously. Under this model, myonuclei would effectively ‘pay in advance’ continuously under the assumption that demand for dystrophin mRNA is always high, and then degrade this mRNA subsequently should it transpire that demand is low. This circumvents the biophysical restrictions of transcribing such a large gene, but the fact nevertheless remains that under most circumstances, demand for dp427 mRNA is indeed low. Under normal, healthy conditions, dystrophin transcription appears to be continuously wasteful for no meaningful benefit. This inefficiency is demonstrably tolerable (given the energetic demands of healthy muscle, perpetual dystrophin transcription likely represents only a tiny fraction of total myofibre metabolism), but given that dystrophin is an ancient gene that predates mammals substantially [16], the continued retention of this system implies it remains of situational use.

As we have suggested previously [8, 11], there are two notable scenarios where dystrophin protein (and thus dystrophin mRNA) might be in high demand: muscle development during embryogenesis and muscle repair following injury. The former establishes myotubes *de novo*, while the latter generates new myotubes to either replace lost fibres, or reconnect undamaged regions of established myofibres (segmental repair). In all these cases, the growing sarcolemma of these nascent myotubes begins in an essentially dystrophin-negative state.

### Muscle injury

Skeletal muscle tissue is highly regenerative: provided the ECM architecture is preserved, muscle can repair completely following even near-total myofibre destruction. Repair is mediated via a dedicated muscle stem cell population termed satellite cells (SCs), which robustly express the paired-homeobox transcription factor *Pax7*. Damage elicits SC proliferation and asymmetric division: one daughter lineage returns to quiescence to maintain the SC pool, while the other proliferates further and migrates to the site of injury [17, 18]. Macrophage-mediated clearance of damaged tissue within the ECM environment occurs early and is essential for efficient repair (demonstrated recently by Collins et al. [19]), affording activated SCs space to proliferate and differentiate into close-packed myoblasts, which align and fuse in a coordinated wave to form long multinucleated myotubes. As myotubes hypertrophy and mature into myofibres, these nuclei remain centrally located, while a second wave of fusion events contributes additional peripheral nuclei [19, 20]. In larger mammals, central nuclei ultimately also migrate to the periphery, but in mice central nucleation persists apparently indefinitely, serving as a permanent histological marker of regeneration (see Fig. 1D). Transcriptionally, repair recapitulates many features of embryonic muscle development, with the same cascade of myogenic basic helix-loop-helix (bHLH) transcription factors (*Myf5*, *MyoD* and *Myogenin*). Similarly, developing myotubes first express embryonic and then neonatal myosin heavy chains (*MYH3* and *MYH8*, respectively), before eventual fibre-type fate choice [21–23].

Dystrophin is expressed at several stages during the repair process, though the temporal and spatial aspects of this expression remain under-characterised. Dp427m is transiently expressed within activated SCs in a spatially-restricted manner that influences early asymmetric division [18], but is not found in proliferating myoblasts (the 16 h transcription is likely mutually exclusive with cell division, as we have noted previously [10]): myoblasts instead express the short isoform, dp71 [24, 25]. Expression of dp427m resumes later, when myoblasts exit mitosis, fuse and differentiate to form myotubes. At this early stage, dystrophin protein is both required but initially absent: a high demand scenario. Demand would remain high as sarcolemmal area increases throughout myotube maturation and hypertrophy, only returning to a basal (low demand) state once muscle repair is complete. We hypothesise that cells might initiate dystrophin transcription early (soon after exiting the mitotic program, prior to any sarcolemmal demands) -the long lag between initiation and completion cannot be circumvented, so early ‘presumptive’ commitment to expression would minimise downstream delays. We further suggest that a system optimised for essentially continuous delivery would ensure that subsequent supply of dystrophin mRNA remains uninterrupted, with control at the post-transcriptional level allowing this supply to be titrated closely to need. Stabilizing completed transcripts would allow dp427 mRNA to accumulate, increasing translational output while demand for protein remains high; when demand returns to low turnover/maintenance levels, surplus mature transcripts (and most future transcripts) could be instead degraded, preventing accumulation of unwanted dystrophin at both mRNA and protein level. In essence, early activation of a strong, “once on, always on” promoter, combined with post-transcriptional fine-tuning, might be the only way to meet (but not subsequently exceed) the high demand in these rare, transient but critical scenarios. In support of this hypothesis, we note that Tennyson and colleagues reported longer transcript half-lives in differentiating myogenic cell cultures [7]; similarly, we typically observe greater numbers of mature mRNAs via smFISH in embryonic myotubes [10, 11]. Dystrophin expression in embryogenesis however involves multiple isoforms, found at different stages and in different tissues. Instead, here we test our theory empirically within a more controlled model system: investigating expression of dystrophin mRNA within injured skeletal muscle throughout the repair process.

## Methods

### Ethics statement

24 wild-type mice (strain C57BL/10J) were used for this longitudinal study, of mixed sexes (9 male, 15 female), all 5–7 months of age. Mice were bred and used under UK Home Office Project Licence PPL 70/7777, approved by the Royal Veterinary College Animal Welfare and Ethical Review Board. All mice were held in open top cages in a minimal disease unit at an average 21 °C in a 12 h light/ 12 h dark light cycle with food and water provided ad-lib.

### Study design and sample collection

Five time points were used to follow muscle regeneration: 2, 4, 7, 14 and 30 days post-injury (DPI), corresponding approximately to: acute degeneration; clearance and activation of the repair programme; early regeneration; late regeneration, and complete repair, respectively. Mice were allocated to sample groups as shown in Fig. 1D (2 DPI, *N* = 4; all other time points, *N* = 5). Assignments were randomised within each sex to avoid uneven allocation of sexes between groups (2 DPI, 1 male, 3 females; all other time points, 2 males, 3 females).

Focal muscle injury was elicited by injection of 20 µl BaCl_2_ (Sigma, 1.2% w/v in sterile saline) into the tibialis anterior (TA) muscle, during general anaesthesia using fentanyl/fluanisone (Hypnorm, Vetapharma, Leeds, UK) and midazolam (Hypnovel, Roche, Welwyn Garden City, UK)) delivered intraperitoneally as published previously [26].

At the appropriate collection time (2, 4, 7, 14 or 30 DPI) mice were killed by cervical dislocation. Muscles (the TA and the adjacent extensor digitorum longus (EDL), both injured and uninjured contralateral) were harvested rapidly post-mortem. Muscles were inspected visually to confirm gross features of injury/on-going repair, then mounted in cryoMbed mounting medium (Bright) on histological corks and rapidly frozen under liquid nitrogen-cooled isopentane. Muscles were mounted in longitudinal orientation in a relaxed state (as described previously [8]). To ensure complete coverage of the damaged region, both the TA and EDL muscles were mounted together. Uninjured contralateral muscles were collected and mounted similarly as controls. All samples were stored at -80 °C until use.

### Cryosectioning

Mounted muscle samples were removed from − 80 °C and transferred to a cryostat (OTF5000, Bright) to equilibrate to cutting temperature. Samples were trimmed by cryostat until the injured region of the muscle belly was fully visible (or to an equivalent depth for uninjured muscles). Longitudinal sections (8 μm thickness) were then collected and mounted on glass slides (Superfrost PLUS) for analysis: sections were collected serially to enable comparison between ISH and immunofluorescent labelling. To preserve RNA integrity for downstream FISH, sections were air dried at -20 °C for 30-60 min before storage in sealed slide boxes at -80 °C. Additional sections were collected (30–40, from the same sectioning depth) into pre-chilled microcentrifuge tubes for RNA isolation.

### Blinding

Given the marked differences in histological appearance between injured and uninjured muscle, blinding to treatment was deemed impractical. To ensure reproducibility, all IHC and ISH staining was conducted in batches containing both treated and untreated samples.

### Histological staining

Haematoxylin and eosin staining was conducted as described previously [27]: slides were equilibrated to room temperature, then immersed in diluted Harris’ haematoxylin (1:1 distilled water) for 3 min. Staining was regressed via acid alcohol, followed by blueing under running tap water for 3 min. Slides were incubated in eosin yellowish (0.5% in water) for 3 min, washed quickly in distilled water and dehydrated through graded alcohols (70–100%). After equilibration to xylene (> 1 h) slides were mounted using DPX (Solmedia).

Acid phosphatase staining used the method described previously [28]: 200 ml sodium acetate staining solution (320mM, pH 4.8) was combined with 4 mg naphthol AS-B1 phosphate (1% stock in dimethylformamide) and mixed well. Separately, 3.2 ml pararosaniline-HCl solution (4% stock in 20% conc. HCl) was mixed dropwise with 3.2 ml sodium nitrite (4% stock in distilled water, prepared fresh), incubated at RT for 2 min, then mixed with the staining solution. pH was returned to 4.8 by careful addition of 1 M NaOH. RT equilibrated slides (as above) were immersed in staining solution at 37 °C for 2–4 h. Slides were washed in distilled water to terminate the reaction, then counterstained (1 min) in Mayer’s haemalum (Sigma), followed by bluing under running tap water for 3 min. Slides were dehydrated rapidly through graded alcohols (~ 30 s per stage) then allowed to dry completely before equilibration to xylene and mounting in DPX as above.

### Immunofluorescence

Immunofluorescence was conducted using slides equilibrated to room temperature (as above). Sections were ringed with hydrophobic barrier pen (ImmEdge, Vector labs) and blocked in 10% goat serum (in phosphate buffered saline supplemented with 0.05% tween-20 (PBS-T)) for 1 h at RT. Primary and secondary antibody incubations were 1 h at RT, with three washes of PBS-T in between each incubation. All antibodies were diluted in PBS-T. Primary antibodies and dilutions used were as follows: dystrophin (rabbit polyclonal Ab15277, Abcam, 1:200); perlecan (rat monoclonal A7L6, Invitrogen, 1:1000); labelling of infiltrating mouse IgG used secondary antibody alone. Secondary antibodies were all AlexaFluor Plus (Thermofisher): anti-mouse 488, anti-rat 555 and anti-rabbit 647 (all at 1:800). Labelling of embryonic myosin used the Zenon labelling system (Thermofisher): antibody (mouse monoclonal MHCd, Novocastra) was preincubated with Alexafluor488-labelled FAb fragments (5 min, RT) followed by blocking with non-specific IgG (5 min, RT): the resultant primary/fluorophore conjugate mix (final primary antibody dilution 1:40) was used alongside secondary antibody labelling, above. Following immunolabelling, nuclei were labelled with Hoechst 33,342 (1:2000, 5 min). Slides were mounted using ProLong Gold antifade (Thermofisher).

### Multiplex FISH

Fluorescence in-situ hybridisation (FISH) was conducted using the RNAscope V2 multiplex labelling system (ACDbio/BioTechne), as described previously [8, 10]. Slides were removed from − 80°C and immediately immersed in chilled (4°C) 10% neutral buffered formalin for 1 hour. Slides were dehydrated through graded alcohols (50%, 70%, 100%), air dried and then baked at 37°C for 1 hour. All remaining steps were according to the manufacturer’s protocols for fresh frozen tissue, including protease treatment to expose mRNA sequence and eliminate any contributions from RNA binding proteins that might affect labelling. Slides were labelled with probes to the satellite cell marker Pax7 (Mm-Pax7-C3, Cat. No. 314181-C3), the cell division marker Ki67 (Mm-Mki67-C3, Cat. No. 416771-C3), and to dystrophin, using the multiplex probe set described previously [10, 11], namely Mm-Dmd (Cat. No. 452801), Mm-Dmd-O1-C2 (Cat. No. 529881-C2) and Mm-Dmd-O2-C3 (Cat. No. 561551-C3), recognising the 5’ (exons 2–10), 3’ (exons 64–75) and middle (exons 45–51) regions of the dystrophin dp427 transcript, respectively. Unless indicated otherwise, all C1 probes were labelled with TSA-Cy3, all C2 probes with TSA-Cy5, and all C3 probes with TSA-Opal 520 (all TSA dyes supplied by Akoya Biosciences). Nuclear labelling used Hoechst 33,342 (1:2000 dilution, 5 min). Slides were mounted using ProLong Gold as above.

### Imaging

Brightfield images (H&E, Acid phosphatase) were collected via a DM4000B upright microscope (Leica Microsystems, Milton Keynes, UK) using either a 10x or 20x objective (HC PL FLUOTAR PH1/PH2, NA = 0.3/0.5, respectively). Individual fluorescence images (ISH, IHC) were collected using a Nikon Eclipse Ni-E microscope (objective: 20x PLAN APO, NA = 0.8) with D-LEDI light source (DAPI/GFP/TRITC/Cy5 filter cubes -Semrock). Whole section fluorescence images were prepared using a DMI6000 (Leica) with a motorised stage, using a EL6000 light source (A4, L5, N3 and Y5 filter cubes -Leica) -multiple images spanning the entire section were collected at 20x (HCX PL FLUOTAR PH2, NA = 0.5), and then background corrected and merged using LASX software (Leica).

### Image analysis

Quantitative analysis of Dystrophin 5’ ISH probe intensity was conducted as described previously [8]: low-exposure (non-saturating) images were collected (3–4 imaging fields per section), and nuclear/sarcoplasmic foci (100–300 of each per image) were then manually defined as regions of interest (ROIs) using ImageJ. Total (background-subtracted) 5’ fluorescence intensity of each ROI was then measured. Sarcoplasmic intensity values (corresponding to individual mRNAs) were used to determine mean per-transcript fluorescence for each ISH experiment: these values were used to estimate corresponding nascent transcript numbers per nucleus. Images were collected from sections prepared from four injured (14 DPI), and four uninjured muscles. A subset of images was also collected from undamaged regions of one injured muscle at 14 DPI.

### RNA isolation and cDNA synthesis

RNA was isolated from frozen muscle cryosections using TRIzol (Invitrogen) as described previously [29], with inclusion of an additional chloroform extraction (1:1) after the phase separation step and inclusion of 10 µg glycogen during precipitation to maximise RNA yield. RNA yield and purity were assessed via Nanodrop (ND1000) and samples with 260/230 ratios below 1.7 were subjected to a second precipitation. All cDNA was prepared using the RTnanoscript2 kit (Primerdesign), using 1600ng of RNA per reaction, with oligo dT and random nonamer priming. For qPCR, 10 µl of each reaction was subsequently diluted 1/20 with nuclease-free water to minimise downstream PCR inhibition. The remaining 10 µl of each reaction was left undiluted to allow accurate quantification of low-abundance dystrophin mRNA via digital droplet PCR (ddPCR).

### qPCR and DdPCR

qPCR reactions were performed in duplicate (reference genes) or triplicate (all others) in 10 µl volumes using PrecisionPLUS SYBR green qPCR mastermix (Primerdesign). 2 µl diluted cDNA was used per reaction (~ 8ng cDNA per well assuming 1:1 conversion), and PCR was conducted in a CFX384 lightcycler, with a melt curve included as standard. All Cq values were determined via linear regression. Primers to reference genes (*ACTB*, *CSNK2A2*, *AP3D1*, appropriate for healthy/damaged muscle, as determined previously [30]) were taken from the geNorm and geNorm PLUS kits (Primerdesign): all give efficiencies of 95–105% and produce single amplicons. Sequences are proprietary but anchor nucleotides and context sequences can be provided on request. Primers to *MYH3* (embryonic myosin heavy chain) and *MYH8* (neonatal myosin heavy chain) were taken from Zhou et al. [31]. Primers to specific regions of the dp427m dystrophin transcript and to dystrophin isoform dp71 (targeting the unique first exon) were those used previously [8, 32], while primers to *SPP1* (osteopontin), *Ki67* and *Pax7* were designed to the corresponding Ensembl sequences using Primer3 (https://primer3.ut.ee/). All primers spanned large introns where possible to avoid amplification of genomic DNA. Primer sequences (where available) are provided below:

MYH3 F 5’-CTTCACCTCTAGCCGGATGGT − 3’.

MYH3 R 5’-AATTGTCAGGAGCCACGAAAAT-3’.

MYH8 F 5’-CAGGAGCAGGAATGATGCTCTGAG − 3’.

MYH8 R 5’-AGTTCCTCAAACTTTCAGCAGCCAA − 3’.

Dp427m Ex1 F 5’-TCTCATCGTACCTAAGCCTCC-3’.

Dp427m Ex3 R 5’-GAGGCGTTTTCCATCCTGC-3’.

Dp427m Ex44 F 5’-TGGCTGAATGAAGTTGAACAGT-3’.

Dp427m Ex45 R 5’-CCGCAGACTCAAGCTTCCTA-3’.

Dp427m Ex62 F 5’-AGCCATCTCACCAAACAAAGT-3’.

Dp427m Ex64 R 5’-ACGCGGAGAACCTGACATTA-3’.

Dp71 F 5’-GTGAAACCCTTACAACCATGAG-3’.

Dp71 R 5’-CTTCTGGAGCCTTCTGAGC-3’.

SPP1 F 5’-ATCTCCTTGCGCCACAGAAT-3’.

SPP1 R 5’-AGCAGTGACGGTCTCATCAG-3’.

Pax7 F 5’-TGCCCTCAGTGAGTTCGATT-3’.

Pax7 R 5’- GAGGTCGGGTTCTGATTCCA-3’.

Ki67 F 5’- ACATTCGTATCCAGCTGCCT-3’.

Ki67 R 5’-GGCTTGCTTCCATCCTCATG-3’.

All data were linearised to relative quantities (RQ), and all genes of interest (GOIs) were normalised to a three-gene normalisation factor (geometric mean of the RQs of *ACTB*, *CSNK2A2*, *AP3D1*). Data were subsequently converted to log_10_(normalised RQ) for statistical analysis and graphical display.

Digital droplet PCR was conducted using 1 µl of undiluted cDNA, as described above (equivalent to ~ 80ng cDNA per reaction, assuming 1:1 conversion). ddPCRs were performed using Evagreen supermix (Biorad), with all primers at 100nM, using a QX200 droplet reader (Biorad). Absolute values were obtained for three regions of dystrophin dp427m and for dystrophin isoform dp71. ddPCR was conducted on a subset of samples, which were calibrated with equivalent sample qPCR Cq values to obtain standard curves for each amplicon. R^2^ values for standard curves were typically > 0.99, allowing derivation of absolute values for the entire sample set (values were then normalised for variation in [cDNA] using the three-gene reference normalisation factor as above).

### Statistical analysis

Nascent transcript numbers within healthy/injured myonuclei at 14 DPI were compared via Welch T test in GraphPad Prism 10.4.2. qPCR data were analysed using a Linear Mixed Model in IBM SPSS Statistics (version 29): treatment group, time point, and time/treatment (interaction) were set as fixed effects. Data were then subjected to post-hoc pairwise analysis and corrected for multiple comparisons via the Holm-Šídák method (using Graphpad Prism). Plots were prepared using Graphpad Prism. Calculations of mature/nascent transcript numbers were conducted using the methodology of Tennyson et al. [1, 7], with the following equation:

$$\:\frac{{5}^{{\prime\:}}}{{3}^{{\prime\:}}}=\frac{nascent+mature}{mature}=\frac{{T}_{transcrip}+{T}_{lifetime}}{{T}_{lifetime}}\:$$

Full transcription of dp427 requires ~ 16 hours from 5’ start to 3’ terminus. Transcription time used for ddPCR primer pairs was thus calibrated based on target sequence position (assuming extension rates of 40bases.sec^− 1^): dp427m exon 1–3 sequence (5’) is fully transcribed ~ 2 h after initiation, while exon 62–64 sequence (3’) is completed ~ 15 h after initiation, thus T_transcrip_ = 13 h for ddPCR. T_lifetime_ is the mean lifetime of a mature transcript: equivalent to transcript half-life divided by the natural log of 2 (i.e.T_1/2_ = T_lifetime_ *0.693).

## Results

### Muscle repair following injury

**Fig. 2:**
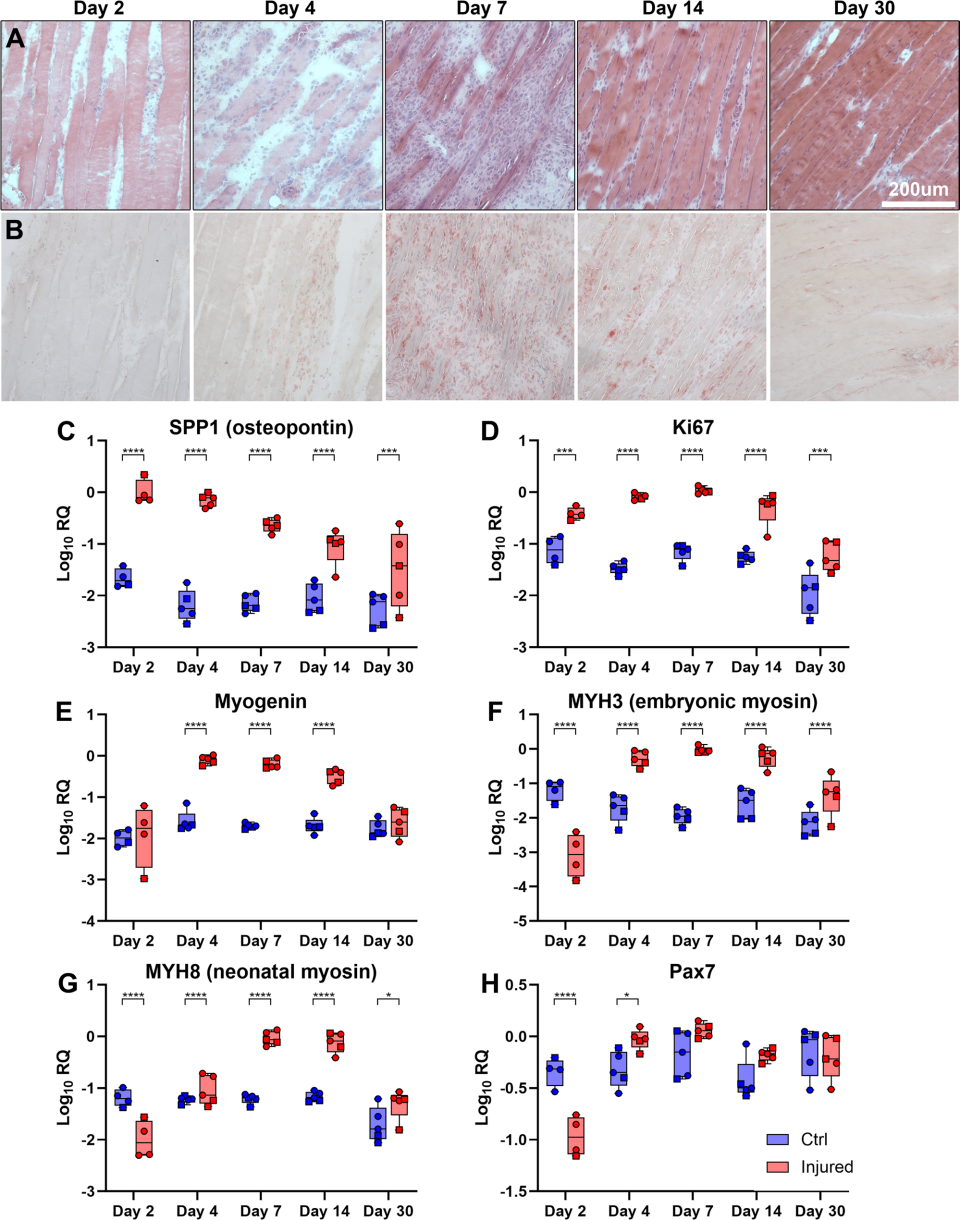
Histological and gene expression analysis of repair timeline shows key regenerative milestones. (**A**) Haematoxylin and Eosin (**H&E**) staining of injured muscle at the indicated days post injury (DPI). At 2 DPI, muscle fibres are pale, fragmented and anucleated, consistent with myonecrosis. By 4 DPI, remaining fibrillar material is being cleared by scavenging mononuclear cells, found both adjacent to, and within, dead fibres. By 7 DPI (early regeneration) nascent multinucleated myotubes are visible, surrounded by large numbers of mononuclear cells. At 14 DPI (late regeneration) myotubes have matured to centrally nucleated myofibres, which continue to hypertrophy. By 30 DPI, the repair process is essentially complete, but regenerated fibres can still be recognised by central nuclei. Staining for acid phosphatase activity (**B**) reveals a peak in staining from 4–14 DPI, consistent with tissue clearance and remodelling during regeneration. Scalebar: 200 μm. (**C-H**) qPCR measurement of gene expression (2 DPI: *N* = 4; other time points: *N* = 5) shows early increases in the inflammatory marker osteopontin (*SPP1*, **C**), with subsequent increases in the proliferative marker *Ki67* (**D**) and the myogenic transcription factor *myogenin* (**E**). Expression of the regeneration-associated embryonic (*MYH3*, **F**) and neonatal (*MYH8*, **G**) myosin heavy chains is consistent with the sequential appearance of these isoforms during regeneration, while expression of the satellite cell marker *Pax7* (**H**) increases modestly during early regeneration. All qPCR analysis used log-transformed RQ values in a linear mixed model, with treatment and time point as fixed variables. There was a significant effect of time (*P* < 0.0001) for all markers, and a significant effect of treatment (*P* < 0.0001) for all markers except *Pax7* (*P* = 0.7). Brackets on individual plots indicate post-hoc multiple comparisons testing between treatment groups at the indicated days post injury (*=*P* < 0.05; **=*P* < 0.01; ***=*P* < 0.001; ****=*P* < 0.0001)

To assess repair status at each time point, we examined muscle sections histologically. Haematoxylin and Eosin staining (Fig. 2A) of muscles at 2 DPI was consistent with acute BaCl_2_ damage: gross myofibre architecture remained, but fibres were hypercontracted and largely devoid of myonuclei, and tissue was oedematous. Rare undamaged fibres were nevertheless present at the periphery of the injury site, and minimal inflammatory cell infiltration was evident. At 4 DPI, cellular infiltration was prominent, with large patches of inflammation-associated mononuclear cells found throughout the tissue, scavenging and clearing damaged myofibrillar material (presumably alongside activated satellite cells and early proliferating myoblasts). By 7 DPI, widespread inflammation remained, but nascent myotubes were also visible, rendered prominent by virtue of their eosin-rich staining and multinucleate character. At 14 DPI, inflammation was markedly reduced while repair was demonstrably underway, with myotubes present in large numbers alongside more mature regenerated myofibres, identifiable by centralised nuclei. In some samples, both centrally nucleated (regenerated) fibres and peripherally nucleated (undamaged) fibres were found in close association, indicating nearly completed repair and restoration of muscle architecture. By 30 DPI, inflammation was minimal and repair was essentially complete, with all regenerated myofibres retaining long chains of central nuclei. Staining for acid phosphatase activity (a lysosome-associated inflammatory/regenerative marker [29, 33]) similarly confirmed these findings (Fig. 2B).

Using qPCR, we also assessed expression of several key genes that characterize aspects of the damage/repair process. mRNA levels of the inflammatory marker *SPP1*/osteopontin (Fig. 2C) were dramatically increased following BaCl_2_ treatment, commensurate with the influx of pro-inflammatory scavenging macrophages: expression at 2 and 4 DPI was almost 100-fold greater than in uninjured contralateral muscles, and though expression subsequently declined, *SPP1* remained modestly elevated even at day 30. Expression within uninjured contralateral muscles was uniformly low throughout, indicating *SPP1* expression was focal, rather than systemic. mRNA for the mitotic marker *Ki67* also increased markedly following injury (Fig. 2D), presumably indicating proliferation of infiltrating macrophage populations. Here, however, expression further increased from day 2 to day 7 (while *SPP1* declined), potentially reflecting a shift in proliferation from scavenging macrophages to myoblasts contributing to muscle repair. Supporting this, expression of the myogenic transcription factor *myogenin* (Fig. 2E), and the regenerative myosin heavy chains *MYH3* and *MYH8* (embryonic and neonatal, respectively, Figs. 2F, G) increased dramatically from day 4 to day 14. *Myogenin* levels were highest at 4 DPI, returning to basal by 30 DPI, while expression of the myosin heavy chains was highest at 7–14 DPI (with *MYH3* increases preceding *MYH8*, consistent with the sequential appearance of these myosin heavy chains during the repair process). Expression of *Pax7* also increased modestly over this period (Fig. 2H), indicating activation and proliferation of the satellite cell population (though this marker was nevertheless low abundance -Cq ~ 25–29). Interestingly, at 2 DPI, levels of *MYH3*, *MYH8* and *Pax7* were lower in injured muscles than in uninjured, possibly reflecting the stark loss of muscle tissue relative to other resident cell populations at this stage.

Taken together, these data were consistent with the histological presentations: acute degeneration and inflammation at 2–4 DPI, commensurate regeneration initiating from day 4–7, established regeneration and repair by 14 DPI, and near-complete repair by 30 DPI (though several gene expression markers remained modestly, but significantly, elevated even at day 30). The overall repair timeline here is slower than that reported by others [19, 22, 23] (possibly reflecting differences in animal age) but otherwise comparable.

### Dystrophin protein expression during repair: immunofluorescence

**Fig. 3:**
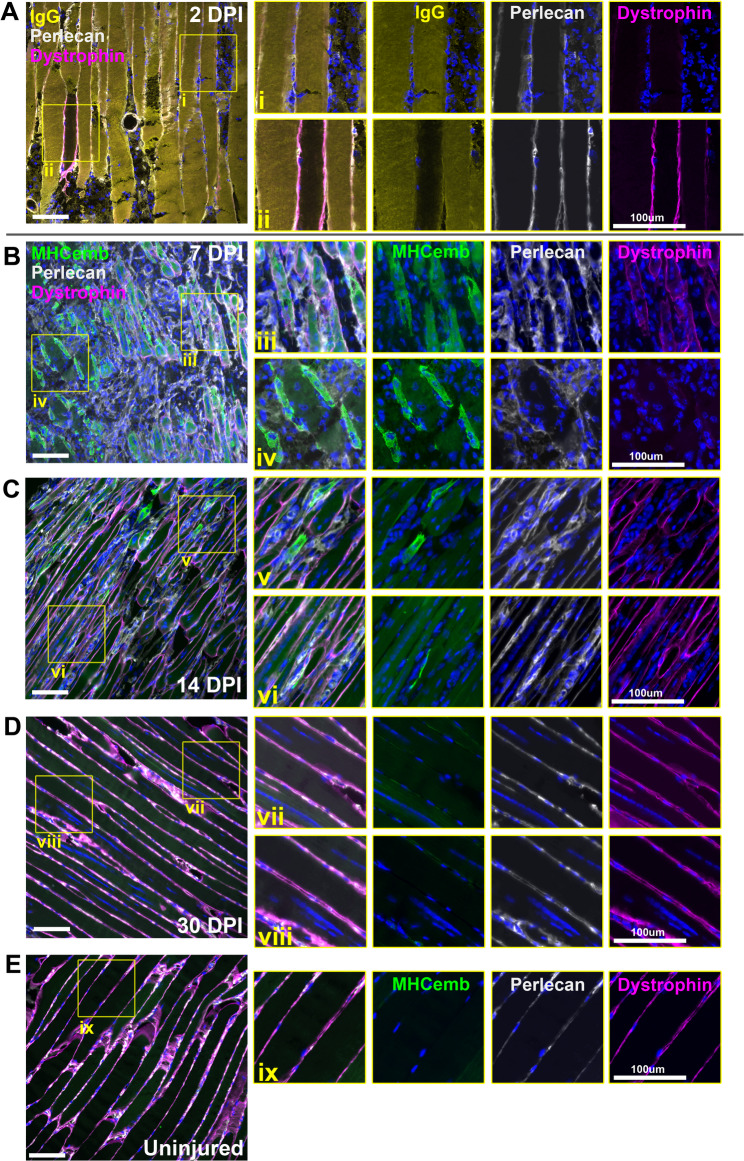
Immunofluorescence of dystrophin protein shows progressive sarcolemmal restoration throughout repair. At 2 DPI (**A**), sarcolemmal dystrophin staining is absent from all except rare, isolated fibres spared damage (inset ii). These fibres concomitantly are negative for infiltrating IgG, which is otherwise widespread (inset i). At 7 DPI, nascent myotubes can be identified by presence of embryonic myosin (**B**). Some myotubes exhibit weak/patchy staining for sarcolemmal dystrophin (inset iii) while others do not (inset iv). By 14 DPI (**C**) robust sarcolemmal dystrophin signal is present in many fibres. The strongest staining is found in more mature fibres that stain only weakly for embryonic myosin signal (inset vi) while fibres retaining strong embryonic myosin staining (younger fibres) show lower dystrophin signal (inset v). By 30 DPI (**D**), strong sarcolemmal dystrophin staining is found in all fibres, at levels comparable to uninjured muscle (**E**). Scalebars: 100 μm

We next used immunolabelling to assess dystrophin protein during muscle regeneration, alongside other protein markers of the repair process: the basement membrane marker perlecan (for underlying tissue architecture); mouse immunoglobulin (for loss of myofibre membrane integrity); embryonic myosin heavy chain (for muscle regeneration). Perlecan revealed myofibre peripheries even during the degenerative/inflammatory phase at 2–4 DPI (Fig. 3A, supplementary Fig. 1), but sarcolemmal dystrophin protein signal within dead/damaged fibres was essentially absent. Similarly, these fibres exhibited marked influx of IgG, indicating loss of membrane integrity (inset panels i). Robust sarcolemmal dystrophin staining was found in rare peripheral fibres negative for sarcoplasmic IgG (i.e. fibres spared BaCl_2_ damage, Fig. 3A, inset panels ii) but dystrophin protein associated with regeneration was absent at these early stages of repair. During active myotube formation at 7 DPI (with concomitant embryonic myosin expression) modest levels of newly produced sarcolemmal dystrophin were observed, though not within all myotubes (Fig. 3B, and inset panels iii and iv). Dystrophin deposition at the myotube membrane was moreover patchy and non-uniform, indicating as-yet incomplete restoration. By 14 DPI (Fig. 3C) sarcolemmal dystrophin levels associated with regenerating (centrally nucleated) myofibres appeared to be approaching healthy levels, with the strongest staining found within fibres staining only weakly for embryonic myosin (more mature fibres -inset panels v and vi). Finally at 30 DPI when the repair process was essentially complete (Fig. 3D), sarcolemmal dystrophin in regenerated fibres was -despite persistent central nucleation- comparable to that within adjacent undamaged fibres (inset panels vii and viii), and to that of uninjured muscle (Fig. 3E). Restoration of sarcolemmal dystrophin protein thus begins early in myotube development (shortly after expression of contractile proteins) and persists throughout the repair process, with levels of sarcolemmal dystrophin progressively increasing as myofibres approach maturity. Over the course of this repair, dystrophin mRNA would presumably be in near-continuous demand, and under our model this demand would be reflected in mature mRNA levels substantially above those found in healthy myofibres.

### Dystrophin mRNA expression during repair: multiplex FISH

**Fig. 4:**
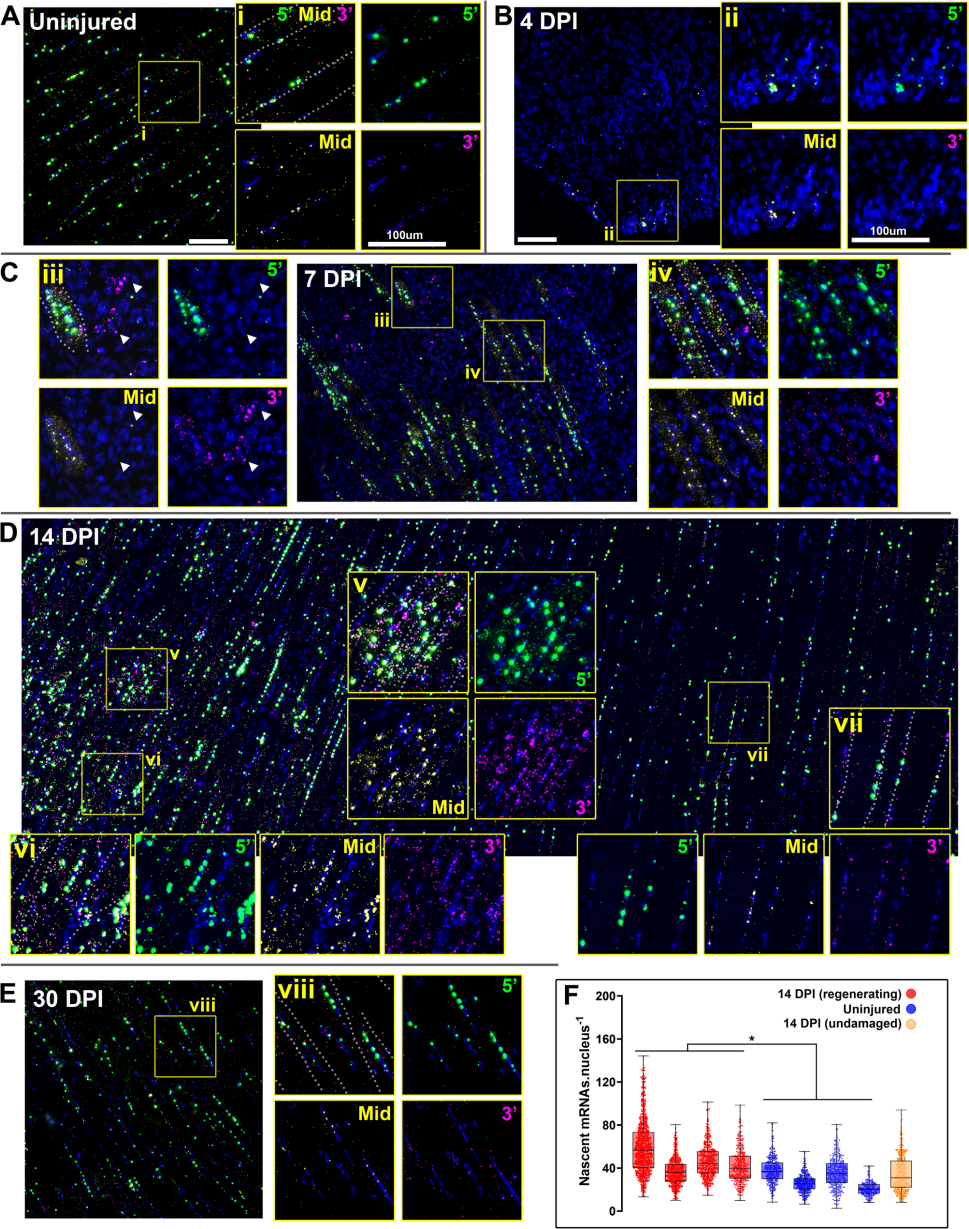
Multiplex ISH of dystrophin mRNA reveals increased transcription and mRNA stability during repair. Multiplex labelling of dystrophin mRNA in uninjured muscle produces a characteristic pattern (**A**): myonuclei are host to large, intense foci of 5’ probe (green), slightly smaller foci of middle probe (yellow), and punctate foci of 3’ probe (magenta), indicating multiplex labelling of nascent dp427m transcripts at different states of maturity. Punctate sarcoplasmic foci of all three probes indicate mature transcripts, found in modest numbers and typically enriched near the sarcolemma (inset i). By 4 days post injury (**B**) dystrophin expression resumes, restricted only to rare patches of nuclei: labelling is predominantly 5’ and middle probe nuclear foci, with minimal 3’ probe labelling (inset ii) indicating nascent transcripts only. At 7 DPI (**C**), myonuclei within nascent myotubes are host to intense 5’ and middle probe foci, indicating increased transcription, while myotube sarcoplasm is richly labelled with all three probes (inset iv) indicating high numbers of mature transcripts. Clusters of nuclei labelled with 3’ probe only can also be observed (inset iii, arrowheads) indicating expression of dp71 in either myoblasts or endothelia. Both nascent and mature dp427 transcript levels remain elevated at 14 DPI (**D**), with strong myonuclear foci and many mature transcripts within growing myofibres (insets v and vi). Dystrophin expression within adjacent undamaged myofibres is however comparable to healthy muscle (inset vii), as is expression at 30 DPI (**E**). Dotted lines on ‘all probe’ insets indicate sarcolemmal boundaries. Myonuclear 5’ fluorescence intensity allows quantification of nascent transcripts per myonucleus (**F**): measurement of four injured muscles at 14 DPI (red) compared to uninjured muscle (blue) or an uninjured region from one injured muscle (orange) reveals myonuclei within regenerating myofibres are host to approximately 50% more nascent transcripts (30–60 per nucleus) than those within healthy or undamaged fibres (20–40 per nucleus). Each datapoint represents a single nucleus (100–300 nuclei measured per muscle). Overlaid box and whisker plot shows median and upper/lower quartiles. *=*P* < 0.05; Welch’s T test using mean per-muscle nascent transcript numbers (uninjured vs. regenerating, *N* = 4). Scalebars: 100 μm

To investigate behaviour of dystrophin mRNA during muscle regeneration, we employed multiplex smFISH (using 5’, middle and 3’ probes as described above, and previously [8, 10]). Labelling within uninjured (contralateral) muscles (Fig. 4A) was consistent with the transcriptional model outlined in Fig. 1C, with a high density of nascent transcripts within myonuclei, and more modest numbers of punctate mature sarcoplasmic mRNAs, predominantly near the sarcolemma. Labelling within injured muscle, conversely, revealed stage-specific differences. At 2 DPI, dystrophin labelling was effectively absent for all three probes (supplementary Fig. 2), consistent with loss of expression within dead/dying myonuclei. By 4 DPI, however, rare dystrophin-positive foci were detectable within small, typically peripheral, regions (Fig. 4B). These foci were nuclear, and near-exclusively labelled with 5’ and middle probe only, indicating nascent, incomplete transcripts: new loci of dystrophin transcription, either within activated satellite cells undergoing asymmetric division, or within myoblasts/early myotubes (absence of mature mRNA also agrees with the lack of detectable dystrophin protein at 4 DPI). Labelling at 7 DPI (when dystrophin protein was detectable within nascent myotubes) was markedly different: intense nuclear signals of 5’ and middle probe were found within the distinctive centrally located myonuclei of newly formed myotubes, indicating high numbers of nascent mRNAs (Fig. 4C), while nuclear 3’ signals remained rare and punctate, indicating rapid export of mature transcripts upon completion. Within the sarcoplasm, numbers of mature mRNAs (indicated by punctate foci of all three probes) were also substantially higher than in uninjured fibres (compare Figs. 4A and C). Small patches of non-myotube nuclei, labelled prominently with punctate 3’ foci only, were also detected (Fig. 4C, inset panel iii, arrowheads) indicating expression of dp71 -potentially endothelia involved in repair-associated angiogenesis, or myoblasts (which transiently express this short dystrophin isoform [24]). By 14 DPI, the central nuclei of maturing myofibres remained strongly positive for nascent transcripts, and the growing sarcoplasm was still richly populated with punctate signals from all three probes (Fig. 4D). This prominent increase in mature transcript numbers was clearly repair-associated: where both regenerating and undamaged fibres could be discerned within the same section, transcript numbers within the latter were modest (Fig. 4D, inset panel vii). Finally, by 30 DPI, when dystrophin signal at the protein level suggested near-complete restoration, transcriptional differences had largely stabilised: myonuclear 5’ and middle probe foci within both peripherally nucleated (uninjured) and centrally nucleated (repaired) fibres were of similar sizes and intensities, and sarcoplasmic transcript numbers were also comparable (Fig. 4E).

Taken together, these findings support our hypothesis: transcription commences early in the repair process (substantially ahead of protein demands, as might be expected), and subsequent mature transcripts appear to be stabilised rather than degraded, consequently accumulating in high numbers while elevated translational demand persists. 5’ and middle probe labelling of nascent mRNAs within immature myofibre nuclei was often more intense than that within uninjured myonuclei, however (particularly at 14 DPI: see Fig. 4D, inset panels v and vi), suggesting greater rates of initiation during regeneration.

To quantify this, we measured fluorescence intensity of nuclear 5’ foci within four of our 14 DPI samples. Comparison with mean intensity of sarcoplasmic 5’ foci (single mature mRNAs) allows estimation of per-nucleus nascent transcript numbers (see methods, and [8]). Numbers of nascent mRNAs within individual nuclei spanned a considerable range (Fig. 4F), as we have shown previously: lower numbers presumably reflect newly initiated transcriptional loci, while higher numbers might include inadvertent measurement of overlapping foci from adjacent nuclei (particularly within inured muscle where many nuclei are closely apposed). Despite this, the bulk of uninjured myonuclei were host to 20–40 immature mRNAs: values that agree with those reported previously [8]. Mean transcript numbers within nuclei of regenerating myofibres were conversely slightly higher (30–60 per nucleus), while nuclei within undamaged regions of injured fibres were similar to those in uninjured muscle. A ~ 50% increase in initiation is modest in transcriptional terms (where larger fold-changes are more typical), but would concomitantly increase mature transcript numbers, potentially explaining our findings. Furthermore, morphology changes might contribute: the sarcoplasmic volume of immature myofibres (and especially nascent myotubes) is lower than that of mature myofibres, and these regenerating fibres are moreover typically host to greater numbers of myonuclei. The high numbers of mature transcripts might not reflect changes in stability, but instead simply be a concentration artefact (i.e. similar transcript numbers within a smaller volume).

### Dystrophin mRNA expression during repair: qPCR

**Fig. 5:**
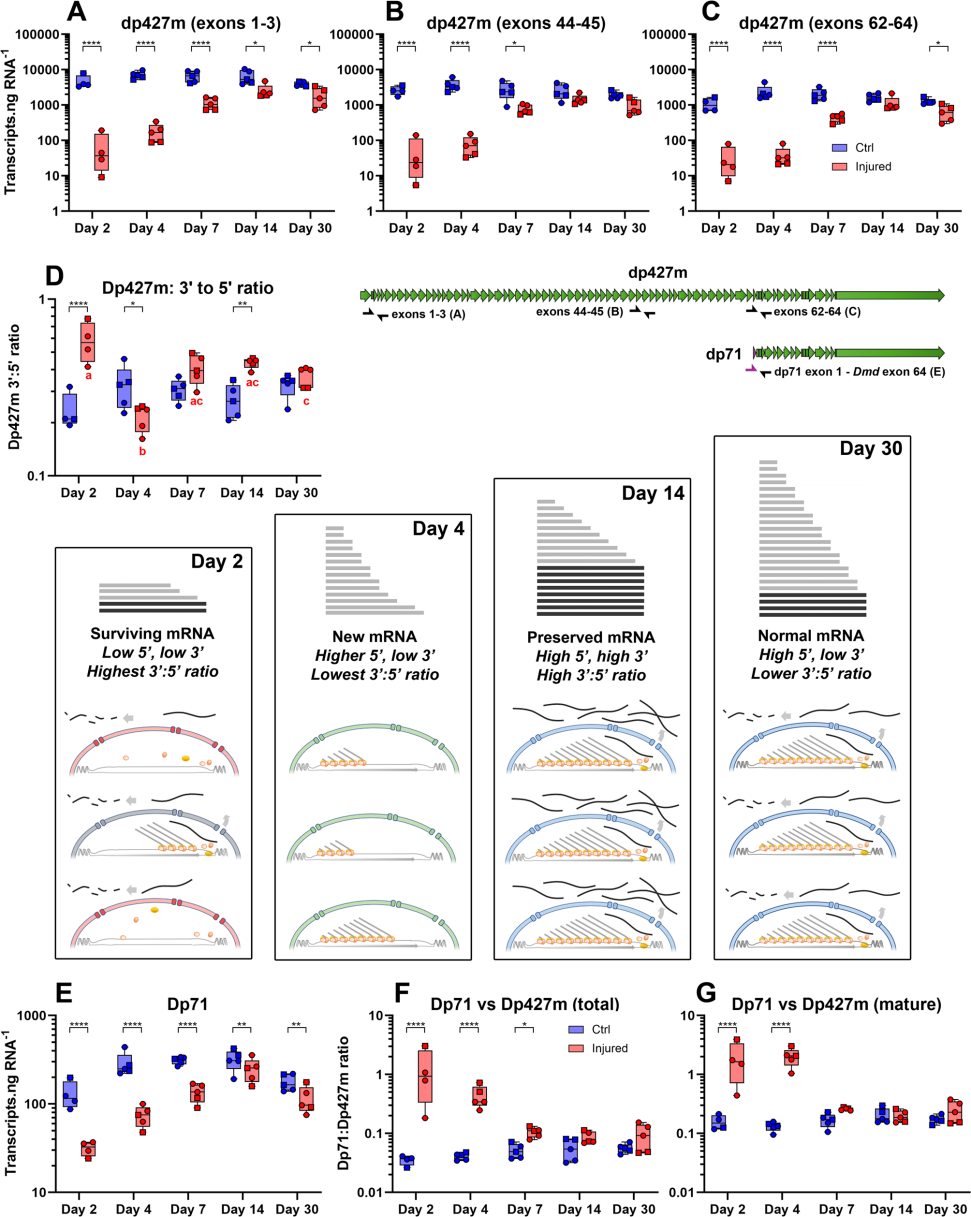
qPCR/dPCR analysis of dystrophin mRNA during repair confirms enhanced mRNA stability during repair. Absolute quantification of dystrophin mRNA in healthy and injured muscles over the course of the repair process, measuring levels of 5’ sequence (exons 1–3, **A**), central sequence (exons 44–45, **B**) and 3’ sequence (exons 62–64, **C**) - see transcript cartoon for approximate locations of PCR primer pairs. In uninjured muscle, transcript numbers remain similar throughout the experiment regardless of sequence region, though individual sequence abundances (5’>>central > > 3’) reflect the ratio of nascent to mature mRNAs (~ 75–80% of total dp427 is nascent). In injured muscle at 2 DPI, levels of dp427 mRNA drop by 2–3 orders of magnitude regardless of sequence position, but at 4 DPI, 5’ and central sequence rise while 3’ does not. By 7 and 14 DPI, levels of all sequence regions increase, but 3’ increases more markedly than 5’ or central sequence. At 30 DPI, transcript numbers return to levels similar to those in uninjured muscle, regardless of region. Expressing 3’ sequence (mature) as a fraction of 5’ sequence (total) highlights these transcriptional changes (**D**). During myonecrosis at 2 DPI, transcription ceases but rare mature dp427m transcripts remain (box ‘Day 2’) -levels of 3’ and 5’ sequence are low, but comparable. At 4 DPI, new transcripts are initiated within nascent myoblasts/myonuclei, but are not yet complete (box ‘Day 4’), giving low 3’:5’ ratios. From 7 to 14 DPI, transcription continues, and mature transcripts are stabilised (box ‘Day 14’) to supply demands, resulting in high 3’:5’ ratios. By 30 DPI demand falls and degradation of mature transcripts resumes (box ‘Day 30’): 3’:5’ ratios fall once more. Expression of the short dystrophin isoform dp71 (using primers targeting the unique first exon -see cartoon) is low in skeletal muscle (**E**), levels decrease further following injury but recover quickly: expressed as a fraction of total dp427m (**F**) or mature dp427m (**G**), this isoform represents only 2–5% of healthy muscle dystrophin, but during early repair accounts for 50% or more of total dystrophin. Brackets on individual plots indicate post-hoc multiple comparisons testing between injured/uninjured muscles at the indicated days post injury (*=*P* < 0.05; **=*P* < 0.01; ***=*P* < 0.001; ****=*P* < 0.0001). For 3’:5’ ratios, post-hoc multiple comparisons between time-points are indicated by letters, where groups sharing a letter are not significantly different: a vs. b: *P* < 0.0001; a vs. c: *P* < 0.05; no uninjured groups were significantly different at any time point)

To address this possibility, and to provide separate objective quantification, we also measured dystrophin transcript numbers via qPCR and ddPCR, using primer pairs targeted to different transcript regions (5’, exons 1–3; middle, exons 44–45; 3’, exons 62–64), in a manner analogous to our multiplex FISH. 5’ sequence -present in essentially all dp427m transcripts- represents total dystrophin, while 3’ sequence represents predominantly mature sequence. The ratio of these sequences thus reflects the fraction of mature transcripts, giving an indirect measure of mRNA stability (at equilibrium this ratio is determined solely by transcription time -an unavoidable 16 h- and mature transcript half-life, not initiation rate: see [1, 7] and methods). Without enhanced stability, a ~ 50% increase in initiation (as detected via ISH) would generate only equivalent increases in mature mRNAs, likely insufficient to explain the numbers of mature transcripts observed. Increases in stability would conversely markedly increase mature transcript numbers, regardless of transcriptional initiation rates. As shown in Fig. 5A, total dp427m expression within uninjured muscle remained unchanged regardless of time point (~ 5,000–10,000 transcripts.ng RNA^− 1^, similar to values reported previously [8]). Levels of middle and 3’ sequence (Fig. 5B, C) were present in lower amounts as expected (reflecting the smaller numbers of transcripts of sufficient maturity to contain middle or 3’ sequence, respectively) but similarly did not change over the course of the experiment.

Expression within injured muscle conversely altered substantially over the repair process: at 2 DPI, total numbers of dp427m transcripts (5’ sequence) fell dramatically (only ~ 10–100 transcripts.ng RNA^− 1^), matching the absence of detectable dystrophin ISH signal at this stage. Levels at 4 DPI were slightly higher (~ 100–500 transcripts.ng^− 1^) and continued to rise during repair, though did not return to uninjured levels even by 30 DPI. Levels of middle and 3’ sequences mirrored these changes broadly, but not exactly, remaining lower at 4 DPI, but also subsequently rising more abruptly: crucial differences suggesting changes in transcriptional behaviour.

To illustrate this clearly, we normalized 3’ sequence (mature) to 5’ (total), correcting for changes in overall dystrophin expression (Fig. 5D) to reveal changes in 3’:5’ ratios during repair. Combining these observations with absolute transcript numbers and ISH data allowed us to build a model of transcriptional dynamics during muscle injury and repair (Fig. 5 panels, Day 2 – Day 30).

Despite high total transcript numbers within uninjured muscle, 3’:5’ ratios were typically 0.2–0.3 regardless of time point, consistent with a mature transcript fraction of only ~ 20–25%, i.e. a ~ 4 h mean lifetime (in agreement with previous reports [7, 8]).

At 2 DPI, the 3’:5’ ratio of injured muscle was markedly higher (near parity in some samples), but total numbers of dp427m transcripts were very low: this reflects the cessation of myonuclear transcription following injury and subsequent myofibre degeneration (Fig. 5 panel: Day 2). Here residual dp427m would be present chiefly as rare, surviving, mature mRNAs (with both 5’ and 3’ sequence), producing the paradoxical increase in 3’:5’ ratio observed.

At 4 DPI the 3’:5’ ratio dropped below healthy levels, even as total transcript numbers began to rise. Following clearance of damaged tissue and initiation of regeneration, dystrophin transcription would begin anew, far from equilibrium and against a ‘blank slate’ background, with little or no mature dp427m mRNA present (Fig. 5 panel: Day 4). Here dystrophin mRNA would thus chiefly be detectable as 5’ sequence but not 3’ (newly initiated transcripts yet to reach completion) and 3’:5’ ratios would fall as observed.

From 7 DPI the ratio reversed again, and by 14 DPI levels of 3’ sequence relative to 5’ were consistently (and significantly) higher in all injured muscle samples, while total dystrophin expression continued to increase. Mature dp427 transcripts would now be present, and in the dystrophin-deficient environment of a newly regenerated myotube/myofibre the demand for these mRNAs would be high (Fig. 5 panel: Day 14): under our model, transcripts would be preferentially preserved, not degraded. This would increase the mature mRNA fraction, giving a higher 3’:5’ ratio than in healthy muscle (as seen). Note that contributions from rare undamaged fibres (which would lower this ratio) cannot be excluded from this data, but the ratios in Fig. 5D nevertheless correspond to a mean mature transcript lifetime of ~ 7–8 h, sufficient to double mature transcript numbers independent of initiation rates (apparent increases in 3’:5’ ratio could also, technically, be transiently achieved by reduced initiation, but both ISH and total transcript numbers show this is not the case).

Finally, as myofibres matured at 30 DPI and sarcolemmal dystrophin protein reached healthy levels, the situation normalised (Fig. 5 panel: Day 30), returning to a 3’:5’ ratio, and total expression level, similar to uninjured muscle.

The small but distinct patches of 3’ probe signals (dp71 expression) observed at 7 and 14 DPI prompted us to measure expression of this short isoform, using a primer pair that includes the dp71 unique first exon. As shown in Fig. 5E, following injury, expression of dp71 fell and then recovered in a manner similar to dp427m. As with 3’ and 5’ sequences, measurement of absolute transcript numbers further allowed these two isoforms to be directly compared across the repair process, with dp71 expressed either as a fraction of total dp427m, or as a fraction of mature dp427m. In uninjured muscle, dp71 transcripts represented only 2–5% of total dp427 (or ~ 10% of mature dp427), regardless of time point. Within injured muscle, reductions in dp71 expression were lower than of dp427m, with the relative proportions of these isoforms consequently changing throughout repair: immediately following injury (2 and 4 DPI), numbers of dp71 and dp427m transcripts were similar (albeit low). Subsequent increases in dp71 expression were swifter (reflecting the short transcription time of this isoform) but also less prominent than those of dp427m: the fraction represented by this short isoform thus remained elevated at 4 DPI, but fell over the course of regeneration. Even by day 30, however, expression appeared modestly elevated over uninjured levels (~ 5–10% of total, ~ 20–25% of mature), suggesting that the repair process was not fully complete even at this stage (supported by other markers of damage/regeneration, above).

### Dystrophin and markers of regeneration: Pax7 and Ki67

To place our work in context within the broader regenerative landscape, we explored spatial expression of dp427 alongside more specific cell markers, combining our 5’ and 3’ dystrophin FISH probes with probes to either the SC marker *Pax7*, or to the proliferation marker *Ki67*.

#### Pax7

**Fig. 6:**
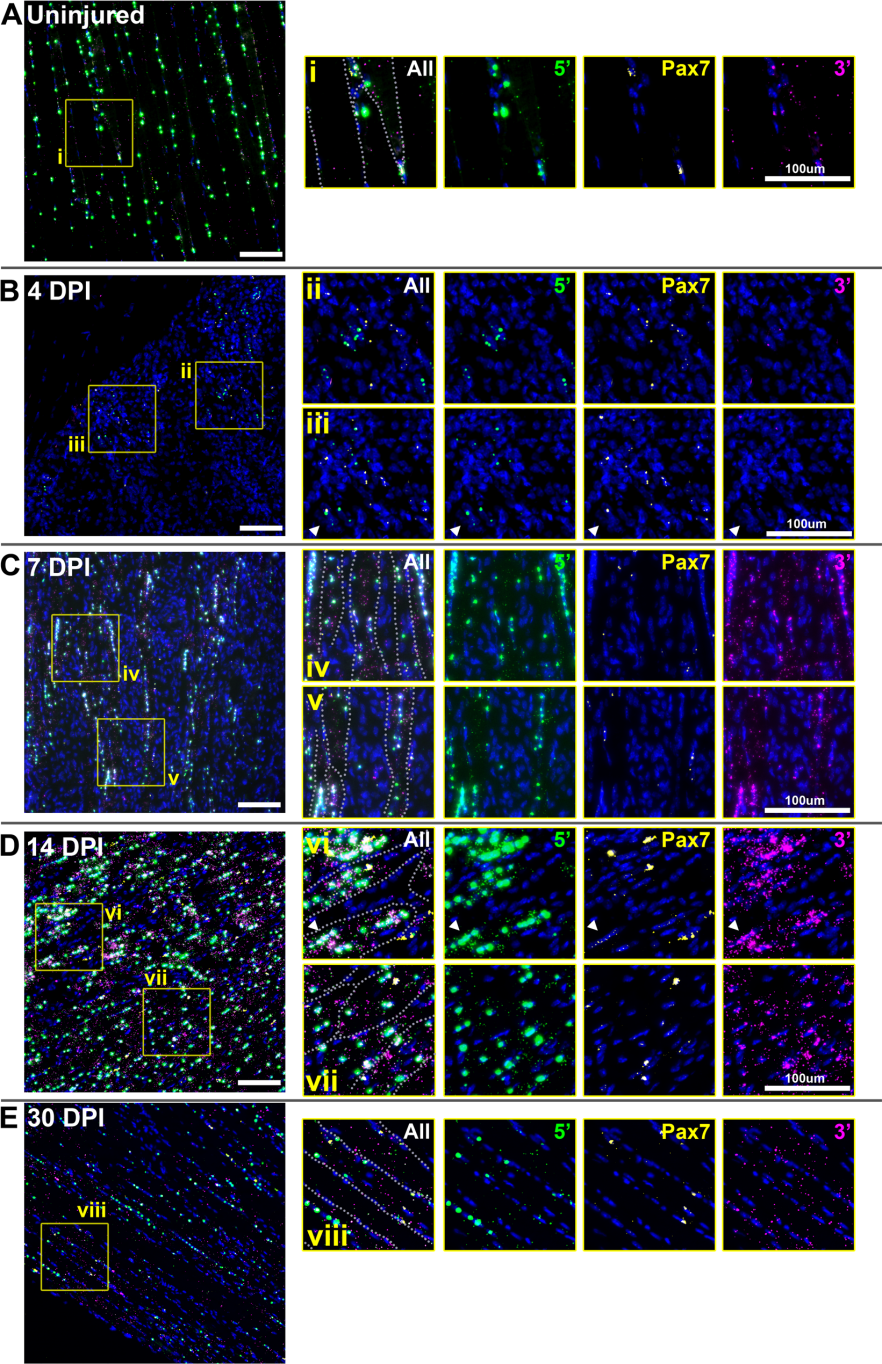
Multiplex ISH of dystrophin and *Pax7* mRNA indicates overlapping expression programs. Muscle labelled with probes for dystrophin 5’ sequence (green) and 3’ sequence (magenta), alongside probes for the satellite cell marker *Pax7* (yellow). In healthy muscle (**A**), *Pax7* expression is found only in rare fibre-adjacent nuclei as expected, apparently accompanied by dystrophin expression in some instances (inset i). In injured muscle at 4 DPI (**B**), *Pax7* expressing cells are typically found in close apposition to sites of nascent dystrophin expression (inset ii). Cells co-expressing dp427 and *Pax7* are rare but detectable (inset iii, arrowheads). Both markers remain in close proximity at 7 DPI (**C**, insets iv, v) though total *Pax7* signal appears modest. At 14 DPI (**D**) intensely *Pax7*-positive nuclei can be found alongside growing myofibres, both with and without co-expression of dp427 (inset vii). Modest *Pax7* expression is also detectable within some robustly dp427-positive myonuclei (inset vi, arrowheads). By 30 DPI (**E**), *Pax7* expressing cells are again found residing peripheral to regenerated myofibres (inset viii). Dotted lines on ‘all probe’ insets indicate sarcolemmal boundaries. Scalebars: 100 μm

Dp427 was expected to partly co-localise with *Pax7* during regeneration, as asymmetric division of SCs first requires transient expression of dp427 [18]. In healthy muscle, *Pax7* labelling was as expected (Fig. 6A), with comparatively high signal found only within distinct, rare, peripheral nuclei consistent with quiescent satellite cells. Some signals co-localised with dystrophin labelling (both 5’ and 3’), implying SCs can transcribe both genes even during quiescence (Fig. 6, inset i). Immediately following injury (2 DPI), we were unable to detect any unambiguously *Pax7* positive nuclei in any samples (supplementary Fig. 3): in agreement with the reduction in mRNA levels determined via qPCR (Fig. 2H) but perhaps unexpected from a regenerative perspective. By 4 DPI, when qPCR indicated a marked increase in *Pax7* expression, patches of *Pax7*-positive cells were found closely associated with sites of nascent dystrophin expression (Fig. 6B). Some cells co-labelled with both *Pax7* and dystrophin 5’ probes (Fig. 6B, inset iii), confirming overlapping transcription of these genes during repair. Similar cells were associated with nascent myotubes at 7 DPI (Fig. 6C). At 14 DPI, the reestablishment of the myofibre architecture rendered *Pax7*-positive cells particularly prominent (Fig. 6D): these were predominantly peripheral, labelling for *Pax7* alone, or both *Pax7* and nascent dystrophin, consistent with the ‘second wave’ of fusion events reported by Collins et al. [19]. We also detected modest *Pax7* signals within strongly dystrophin-positive central nuclei of some maturing myofibres, suggesting that *Pax7* transcription continues post-fusion to some extent (Fig. 6D, inset vi). By 30 DPI, *Pax7* was once again restricted to rare, peripheral nuclei, consistent with completion of repair (as with uninjured muscle, a degree of co-localisation with dystrophin 5’ and 3’ probes was observed -figure 6E).

#### Ki67

We have previously argued that dp427 might be largely restricted to post-mitotic populations purely due to incompatibility of a 16-hour transcription time with concomitant active DNA replication (the genetic locus cannot be transcribed and replicated simultaneously) [8, 10, 11]: accordingly, we did not expect *Ki67* to co-express with dystrophin. In uninjured samples *Ki67* probe signal was essentially absent (supplementary Fig. 4), consistent both with the low measured expression via qPCR (Fig. 2D), and the minimally mitotic nature of mature skeletal muscle.

**Fig. 7:**
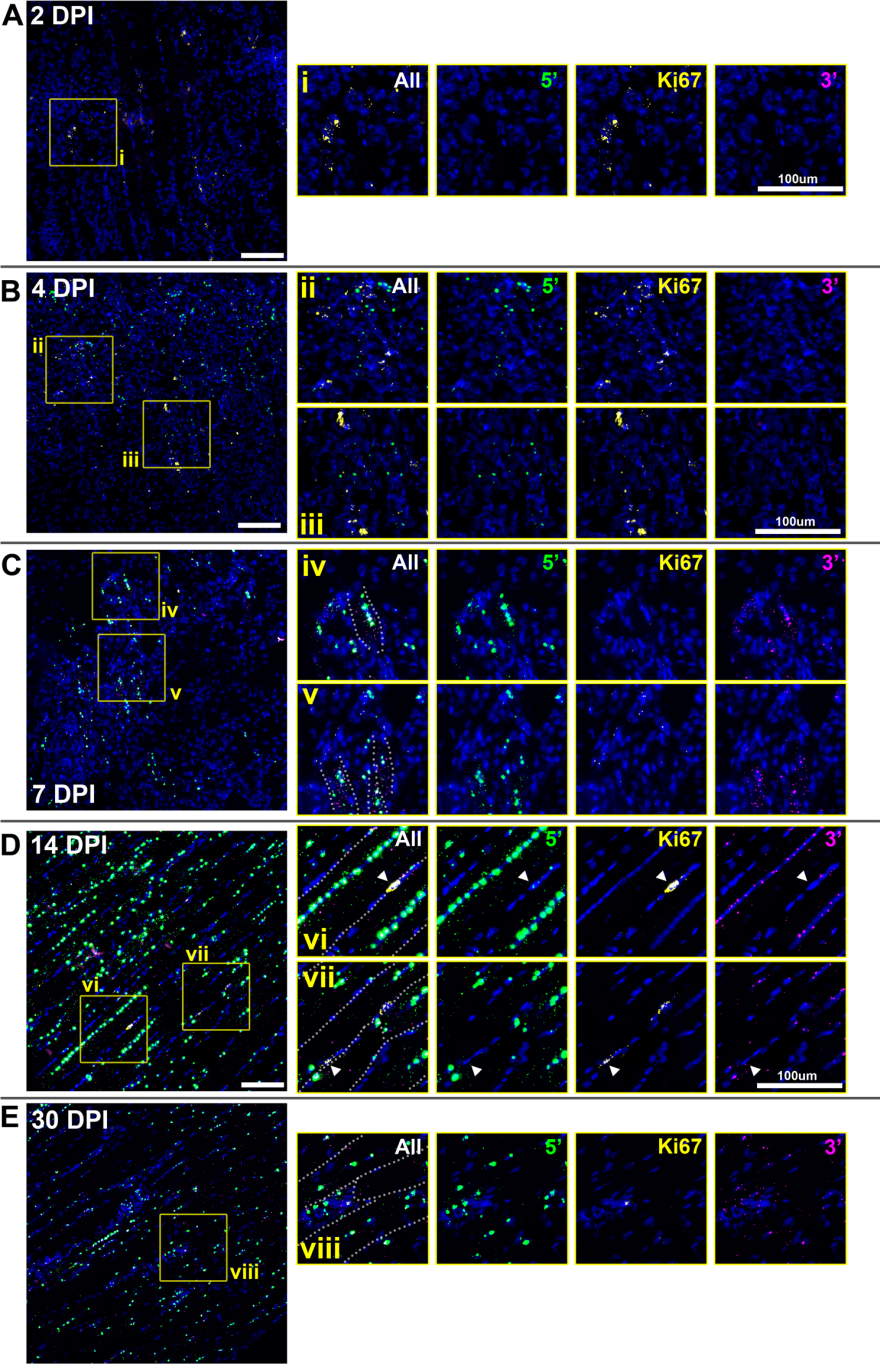
Multiplex ISH of dystrophin and *Ki67* mRNA reveals rare co-expression. Muscle labelled with probes for dystrophin 5’ sequence (green) and 3’ sequence (magenta), alongside probes for the cell division marker *Ki67* (yellow). *Ki67* was detectable even at 2 DPI (**A**) when dp427 expression was absent, but expression was restricted to focal clusters of cells (inset i) rather than widespread. Focal *Ki67* clusters were also evident at 4 DPI (**B**), found close to patches of nascent dp427 expression, but no co-expression was detected (insets ii, iii). At 7 DPI (**C**), *Ki67* expression was restricted to rare cells found in close proximity to some, but not all developing myotubes (insets iv, v). By 14 DPI (**D**), ki67 was found only in peripheral (rather than central) nuclei (inset vii, arrowheads), which were negative for dp427 in all except one instance (inset vi, arrowheads) where prominent *Ki67* expression was found alongside punctate 5’ probe signal indicative of recently initiated dp427 transcription. At 30 DPI (**E**), rare *Ki67*-positive nuclei could be detected at fibre boundaries, in some instances co-localising with or expressed adjacent to 3’ probe signal indicating dp71 (inset viii). Dotted lines on ‘all probe’ insets indicate sarcolemmal boundaries. Scalebars: 100 μm

qPCR suggested marked increases in *Ki67* expression following injury (Fig. 2D), however ISH revealed that even within damaged, actively regenerating tissue, expression of *Ki67* was not widespread. At 2 DPI (when dystrophin expression was absent) *Ki67* positive signal was restricted to isolated patches of mononuclear cells (Fig. 7A). At 4 DPI this focal distribution persisted (Fig. 7B): here, as with *Pax7* (above), *Ki67*-positive cells were often found in close association with sites of nascent dystrophin expression, but no unambiguously double (*Ki67*/dp427) positive cells were observed. *Ki67* positive cells were similarly detected at 7 DPI, typically close to some, but not all, nascent myotubes (Fig. 7C, insets iv, v). Despite qPCR data indicating peak *Ki67* expression at this stage, ISH signal remained sparse. By 14 DPI, when muscle architecture was reestablishing, *Ki67* expression was restricted to distinct, individual nuclei located along the periphery of maturing myofibres (Fig. 7D). Some of these nuclei were also associated with 3’ probe alone (dp71: consistent with proliferating myoblasts [24]), but in one instance we detected clear co-expression of *Ki67* and nascent (5’ probe only) dystrophin expression (Fig. 7, inset vi, arrowheads), indicating that unexpectedly, proliferation and dp427 expression might partially overlap. By 30 DPI (Fig. 7E), *Ki67* expression was restricted to rare individual nuclei (2–3 per entire muscle section) neither within, nor peripheral to myofibres, but associated with nuclear-rich clusters that were also often 3’ probe positive (possibly dp71-expressing vascular endothelia).

Collectively, these data support and refine the regenerative timeline, indicating that early (day 2) proliferation is associated with non-myogenic cells, while subsequent sites of proliferation are chiefly associated with establishment of nascent myotubes and hypertrophy of maturing myofibres.

## Discussion

Expression of full-length dystrophin (dp427) is by necessity unusual: the 16-hour transcription time enforced by the enormous size of the *Dmd* gene precludes regulation via transcriptional initiation over any smaller timescale. Myonuclei instead initiate dystrophin transcription continuously and control mRNA levels post-transcriptionally, a wasteful but responsive ‘pay in advance’ model that circumvents this delay, allowing responsive control. Healthy muscle demands are however low: most mature transcripts are degraded shortly after completion, and immature transcripts represent 70–80% of total dystrophin mRNA, indicating marked oversupply. Fine control over expression might not only be wasteful, but unnecessary. Dp427 is also associated almost exclusively with terminally differentiated, post-mitotic cell types (principally skeletal, smooth and cardiac muscle, and neuronal lineages within the brain), lineages with differentiation processes that require multiple days, and with long-term dystrophin demands that are both relatively low and stable. Even with a 16-hour delay, dystrophin mRNA could presumably be continuously supplied at levels sufficient to meet these modest demands. Oversupply of dp427 transcripts is nevertheless a conserved phenomenon, found in both muscle and neuronal lineages, and across multiple species (mice, dogs and humans), suggesting that responsive expression remains essential (i.e. changes in demand can occur). Here we investigated dystrophin expression at both the mRNA and protein level during BaCl_2_-induced skeletal muscle injury and repair, covering key stages in the repair process over which demand for dystrophin was expected to change substantially: our findings support our transcriptional model but also reveal further insights into transcriptional control.

The near-complete loss of dystrophin mRNA at 2 DPI was expected, given the destruction of most myofibres. Similarly, while dead/damaged myofibres could be delineated via basement membrane perlecan staining, dystrophin protein was absent, indicating loss/degradation of the sarcolemma. This absence of detectable protein persisted at 4 DPI, when rare patches of nuclear dystrophin 5’ probe signal (and increases in 5’ sequence via qPCR) were detected, allowing us to attribute these foci unambiguously to recent transcriptional initiation. Macrophage-mediated clearance of cellular debris is essential for myotube formation [19], and at 4 DPI this scavenging was ongoing, explaining the absence of recognisable myotubes at this stage: this nascent dp427 mRNA instead appeared to be associated with mononuclear cells, and was predominantly (but not exclusively) distinct from cellular *Pax7* expression, implying most early expression occurred within myoblasts/myocytes rather than SCs. Use of a *Ki67* probe allowed us to assess proliferation within these cell populations, something we previously argued might be mutually exclusive with dp427 (but not dp71) expression [10, 11], given the conflicts between DNA replication and a 16-hour transcription process. Our data here suggest this hypothesis merits revision: in at least one instance (Fig. 7D) the proliferation marker was unambiguously and prominently found alongside nascent dystrophin (small nuclear foci of 5’ probe only), demonstrating overlap can occur. *Ki67* expression chiefly increases during G_2_/M, rather than S-phase [34]: dp427 expression might thus only be precluded during S-phase, accelerating transition from proliferative to dystrophin-expressing status. Alternatively, expression might be independent of cell cycle status, but simply prematurely terminated by DNA replication, limiting successful dystrophin expression only to periods outside S-phase (given the inherently wasteful nature of basal dystrophin expression, such inefficiency is not implausible). Collectively, these data suggest that nascent dp427 mRNA expression might begin remarkably early in the myogenic differentiation process, potentially coincident with exit from the cell cycle, prior to myoblast fusion. As the delay between initiation and completion must be endured at least once, committing to expression in advance of fusion would render nascent myotubes essentially ‘primed’ for completion, with the price largely paid prior to overt demands at the sarcolemma.

The following regeneration stages (7 and 14 DPI) best demonstrated these sarcolemmal demands, from formation of nascent myotubes through to maturing myofibres. Here sarcolemmal dystrophin protein was detectable, but not yet fully restored, with the continuous hypertrophy of these maturing fibres (and concomitant expansion of sarcolemmal surface area) serving to keep demand elevated. Exactly as we predicted, high demand resulted in marked increases in mature transcript numbers, and specific analysis of 3’:5’ ratios suggested that this was achieved by increased mRNA stability. Numbers of nascent mRNAs (as represented by intensity of nuclear 5’ labelling) also increased, however, implying that demand is controlled at multiple levels: over short timescales via stability, and longer periods by supply. In line with this, at 30 DPI when repair was near-complete, both stability and supply returned to near-normal levels.

We also assessed dp427 expression within centrally located myonuclei (which necessarily supply all initial dystrophin demands within myotubes and immature myofibres until secondary fusion events contribute peripheral nuclei). Persistent central nucleation is a mouse-specific phenomenon, and while it does not appear to be deleterious, this central location (surrounded by dense myofibrillar apparatus) might impair appropriate targeting of exported mRNAs, preventing nuclei functionally contributing to myofibre transcription. Central nuclei continue to be robustly transcriptionally active, however [35], and we have demonstrated nascent dystrophin expression within central nuclei of *mdx* mouse fibres [8], though degradation of completed *mdx* dystrophin transcripts via nonsense-mediated decay (NMD) precluded assessment of mature mRNA behaviour. As we show here, in injured WT muscle, high numbers of both nascent and mature transcripts were associated with central nuclei at both 7 and 14 DPI (Fig. 4C, D), confirming that these nuclei contribute the bulk of dystrophin mRNA to the growing sarcolemma. This expression persisted at 30 DPI, with scattered mature transcripts found both close to central nuclei and distributed throughout the sarcoplasm (Fig. 4E), suggesting that even after addition of multiple peripheral myonuclei, these central nuclei continue to contribute dystrophin transcripts, and can target them to the sarcolemma.

The changes in dystrophin demand explored here admittedly represent extremes: myofibre destruction and concomitant compensatory regeneration can be induced and followed experimentally, but will not be commonplace under normal conditions (though segmental necrosis can occur following exercise [36, 37]). Milder insults such as membrane microtears and disruptions likely occur on a more regular basis, however: these might not require SC activation, but their repair would nevertheless elicit changes in demand. Membrane repair is multiphasic [38]: a ‘patch’ forms within seconds (sealing the membrane and preventing toxic calcium flux), but subsequent ‘full’ repair of the membrane occurs more slowly, with the precise pathway further varying with extent of damage (including myonuclear migration [39]). Restoration of sarcolemmal dystrophin occurs during this second phase: under the model proposed here, local dp427m transcripts would be available immediately, and selectively preserved, allowing restoration to begin promptly.

Questions undoubtedly remain: our previous work has explored the ‘what’, demonstrating that dp427 ‘transcript imbalance’ results simply from continuous production, lengthy transcription and subsequent rapid degradation, such that most transcripts are nascent rather than mature. Our study here provides a framework for the ‘why’, showing that this counterintuitive system allows for rapid and fine control over mature mRNA levels across states of varying demand during muscle repair. The ‘how’, at present, remains under characterised.

**Fig. 8:**
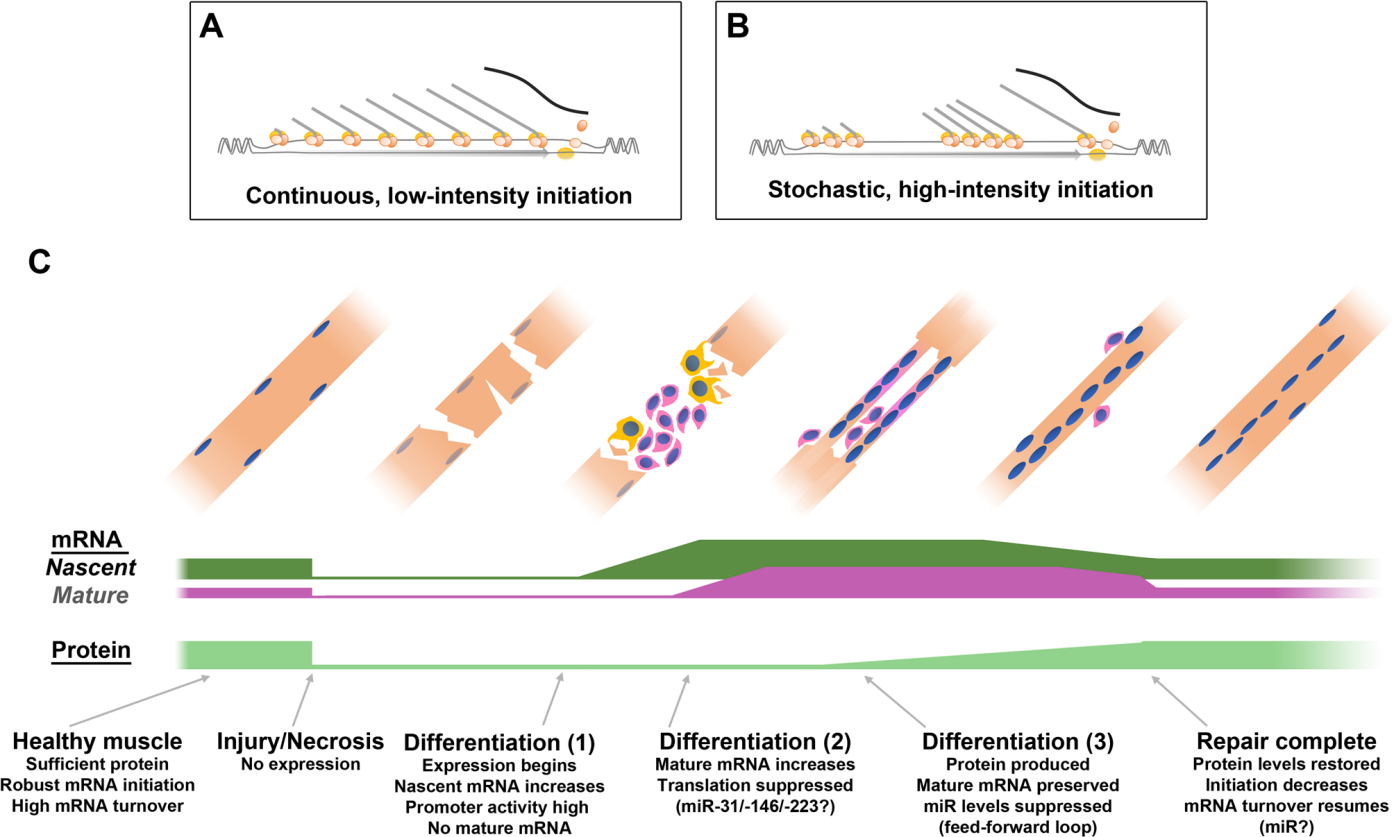
Regulation and dynamics of dystrophin expression. Possible transcription modes for dystrophin expression: constitutive (**A**), where transcripts are initiated continuously at low levels, distributed along the *Dmd* locus; ‘bursty’ (**B**), where high numbers of initiation events occur at stochastic intervals, leading to ‘transcriptional bubbles’ along the *Dmd* locus, containing many transcripts of similar maturity. Timeline for dystrophin during muscle repair (**C**): in healthy muscle transcript degradation is rapid, and nascent mRNAs outnumber mature, while protein levels remain high and stable. During myonecrosis levels of nascent and mature mRNA, and protein all drop to near-zero. Early in differentiation, transcription begins, generating nascent mRNA numbers that surpass normal expression. As differentiation proceeds, transcripts reach maturity and are selectively preserved rather than degraded. Translation is initially suppressed through microRNA interactions, but low levels of dystrophin protein then suppress miR expression and potentiate further translation in a feed forward loop that generates sufficient dystrophin protein to supply the sarcolemma during hypertrophy. Once repair is complete, mRNA degradation resumes, and most dp427 mRNA is again nascent, not mature

Both initiation rate and mRNA stability can influence transcript levels, and while we do not directly measure mRNA turnover here, our data suggests stability is the principal mediator of short-term tuning. Nevertheless, increases in dp427 transcriptional initiation clearly also occur during regeneration. The increases measured here were modest, however (~ 50% over basal, or ~ 40–60 nascent transcripts per nucleus), implying that even under peak demand, output cannot be markedly enhanced. As ~ 60 active transcription complexes are unlikely to represent maximum occupancy of a 2.3 Mb locus, initiation itself might instead be limiting (as suggested by others [4]), either reflecting constitutive but slow assembly of initiation complexes, or stochastic but high-efficiency transcriptional ‘bursts’ (Fig. 8A, B) as found in many genes [40, 41].

Post-transcriptional regulation of mature mRNA stability over short timescales, conversely, implies similarly responsive control mechanisms. Stability can be mediated via the 3’ UTR [42], which in dystrophin is ~ 2.6 kb, well conserved [43], and constitutively present in dp427 (as well as other isoforms, such as dp71). Long 3’ UTRs are inherently vulnerable to degradation [44], and also interact with RNA binding proteins (RBPs), which can mediate stability (the RBP vigilin binds the dystrophin 3’ UTR, but its role is unclear [45, 46]). The dystrophin 3’ UTR has moreover been shown to destabilise mRNA in myoblasts, but not in maturing myotubes [47].

Muscle mRNAs can also be transported, in a microtubule-dependent fashion [48]: the preferential localization of mature dp427m transcripts near the sarcolemma, whether produced by peripheral or central nuclei, supports such targeting (interestingly, dp427 protein also interacts with tubulin [49]). This targeting does not appear to vary with demand, however, arguing against a role in turnover. Translation also influences stability: translation and degradation factors compete for access to polyA tails [50], thus suppression of translation (for example, by microRNAs) might inherently promote degradation. MicroRNAs play several roles in myogenesis [51, 52] and some (including miR-31, -146 and − 223) can bind the dystrophin 3’ UTR and inhibit translation [53, 54]: whether levels of these miRs alter during repair progression remains unclear (microRNA expression during early stages of muscle regeneration has been studied [55, 56], but later stages of the repair process are poorly characterised).

How myofibres sense dystrophin levels is another puzzle: sarcolemmal dystrophin itself plays multiple signalling roles [57], making it an obvious candidate for fibre-wide ‘global’ sensing, but whether this would suffice for more local scenarios (such as repair of membrane microtears) remains unclear. Microtears cause transient but dramatic Ca^2+^ influxes [58], and aberrant sarcolemmal calcium alone can elicit Duchenne-like pathology [59], thus Ca^2+^ mediated modulation of ‘local’ post-transcriptional programs is plausible. Further studies are needed to address these questions.

A final question is simply: why does the mammalian dystrophin gene remain so enormous? The regulatory systems described here all stem from the size of the dystrophin locus, yet only ~ 0.5% of the *Dmd* gene is coding sequence: the remainder is intronic, and thus potentially dispensable. Indeed, while coding sequence is well conserved across lineages, gene length is less so. Mammalian dystrophin genes are ~ 2-2.3Mb [60], but those of birds and amphibians are only ~ 1.1 Mb (~ 8 hour transcription time), while fish and invertebrate dystrophins are a mere 30-300 kb in size (short enough to be transcribed in less than 2 hours) [61, 62]. This intronic expansion has resulted in novel promoters (and thus isoforms [61]), and the dystrophin isoform milieu of mammals is certainly more complex than other metazoans, but direct comparison of size suggests that expression is problematic only within the tetrapods. The transcriptional regulation explored here might have co-evolved to accommodate this expanding length, but might consequently also necessitate such enormity: subsequent reductions in gene size would deliver mature transcripts more rapidly, disrupting the balance of oversupply and post-transcriptional control. A corollary of this is that we would expect our transcriptional model to apply to all tetrapod dystrophin expression, but not necessarily to fish, or to arthropods. Supporting this, zebrafish dystrophin expression differs substantially from mammals [63], without prominent nuclear nascent transcript numbers [64]. Future work assessing 5’:3’ ratios in these lineages might confirm these findings.

## Conclusion

The size of the mammalian dystrophin gene renders expression perennially problematic, with conventional regulation taking too long to deliver mRNA at times of need, yet al.so risking marked oversupply once demands are met: either never enough, or always too much. The work here builds toward a picture whereby myonuclei pay the 16-hour price both in advance and in perpetuity, solving the problem instead, at almost every stage, post-transcriptionally. Expression begins early to avoid delay, but translation is suppressed (likely via microRNA interaction, as shown by others [53, 65]) to prevent aberrant protein accumulation prior to myotube formation. Subsequent high sarcolemmal demands are addressed by relieving translational suppression and enhancing transcript stability, ensuring large numbers of translation-competent mRNAs. Once myofibres reach maturity and demand falls, supply remains high but turnover increases to keep transcript levels low while retaining capacity for rapid responses should they be needed in future (Fig. 8C).

Finally, while we studied healthy tissue here, these mechanisms will also likely be present in dystrophic muscle. The asynchronous nature of dystrophic regeneration presumably hampers stage-specific control, as does dysregulation due to persistent dystrophin deficiency (shown for miR-31 [65]), but despite these factors, dystrophic myonuclei demonstrably continue to produce dp427 mRNA. This has therapeutic implications: achieving high levels of corrected or recombinant dystrophin mRNA is a major challenge to current approaches. Relieving translational suppression enhances dystrophin protein levels following exon skipping [53], and our work here implies additional regulatory systems exist, all of which might be therapeutic targets: pharmacological modulation of mRNA turnover and translation might therefore be a means to achieve beneficial protein restoration with even low efficiency correction at the mRNA level.

## Supplementary Information

Below is the link to the electronic supplementary material.

**Supplementary Material 1**: **Supplementary Fig. 1**: Dystrophin protein remains absent at 4 DPI. At 4 DPI, fibre profiles can be identified via perlecan staining, but sarcolemmal dystrophin staining is essentially absent from all damaged fibres (which are concomitantly robustly positive for infiltrating IgG). Scalebars: 100µm

**Supplementary Material 2**: **Supplementary Fig. 2**: Dystrophin ISH signal is absent at 2 DPI. Multiplex labelling of dystrophin mRNA (5’: green; middle: yellow; 3’: magenta) in injured muscle at 2 days post injury reveals no labelling with any probe, indicating absence of both mature and nascent transcripts.

**Supplementary Material 3**: **Supplementary Fig. 3**: *Pax7* expression is not detected at 2 DPI. Multiplex labelling of dystrophin and *Pax7* mRNA (dp427 5’: green; dp427 3’: magenta; *pax7*: yellow) in injured muscle at 2 days post injury reveals no labelling with any probe, indicating absence of nascent and mature dp427 transcripts, and no detectable *pax7* expression.

**Supplementary Material 4**: **Supplementary Fig. 4**: *Ki67* expression is minimal in healthy muscle. Multiplex labelling of dystrophin and *Ki67* mRNA (dp427 5’: green; dp427 3’: magenta; *ki67*: yellow) reveals the characteristic strong nuclear 5’ signals of nascent dp427 mRNAs and the punctate sarcoplasmic 5’ and 3’ signals of mature transcripts, while Ki67 labelling is very low and highly sporadic, found only within rare individual nuclei.

## Data Availability

qPCR/ddPCR data generated for this study, and downstream statistical analysis, are available at the following figshare repository DOI: 10.6084/m9.figshare.28869476Images used for myonuclear transcript quantification, and downstream analysis, are available at the following figshare repository DOI: 10.6084/m9.figshare.28869500Images (ISH, IHC) collected for qualitative analysis and preparation of figures, and whole-section scans, are available from the corresponding author on reasonable request.

## Abbreviations

dp427: Dystrophin protein 427 kDa
DAGC: Dystrophin associated glycoprotein complex
ECM: Extracellular matrix
DMD: Duchenne muscular dystrophy
smFISH: Single-molecule fluorescence in situ hybridisation
SC: Satellite cell
BaCl_2_: Barium chloride
bHLH: Basic helix-loop-helix
DPI: Days post-injury
TA: Tibialis anterior
EDL: Extensor digitorum longus
NMD: Nonsense-mediated decay
miR: MicroRNA
nNOS: Neuronal nitric oxide synthase
