# Supplementary figures and images for: Spatiotemporal analysis of dystrophin expression during muscle repair
Source: Skelet Muscle. 2025 Oct 2;15:27. doi: 10.1186/s13395-025-00398-y (PMC12492914; doi:10.1186/s13395-025-00398-y)

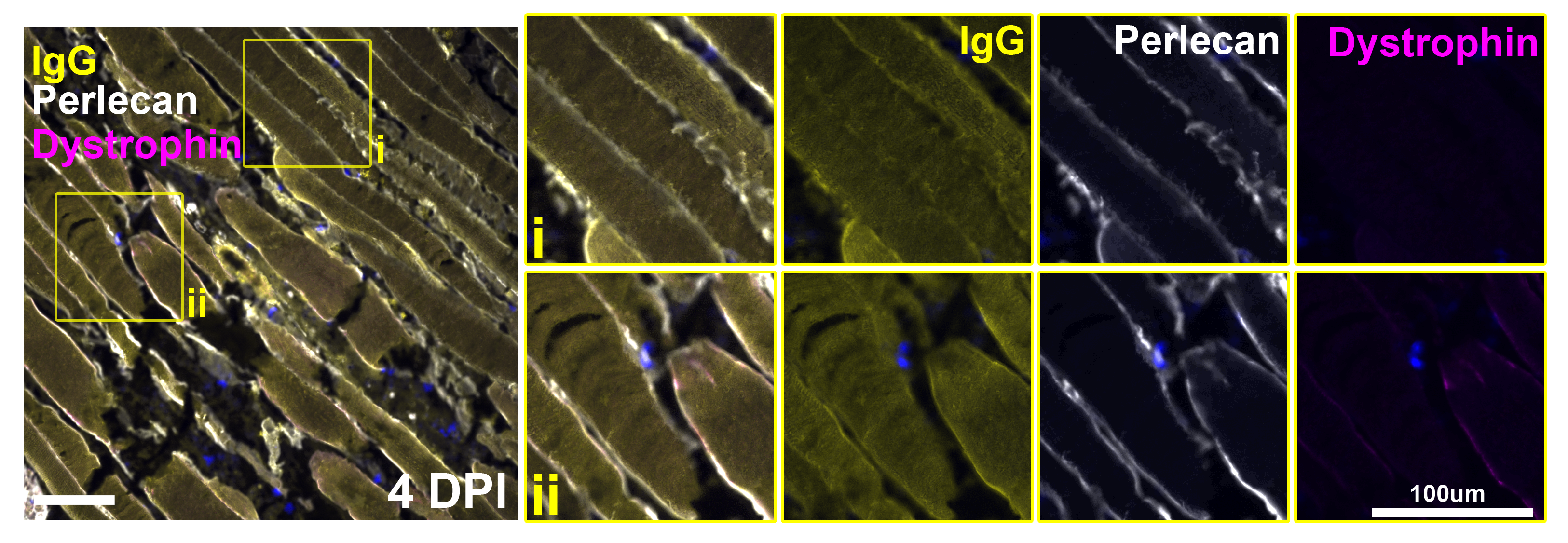

Supplement: Supplementary file 1 — Supplementary Material 1: Supplementary Fig. 1: Dystrophin protein remains absent at 4 DPI. At 4 DPI, fibre profiles can be identified via perlecan staining, but sarcolemmal dystrophin staining is essentially absent from all damaged fibres (which are concomitantly robustly positive for infiltrating IgG). Scalebars: 100µm [file 13395_2025_398_MOESM1_ESM.tif]

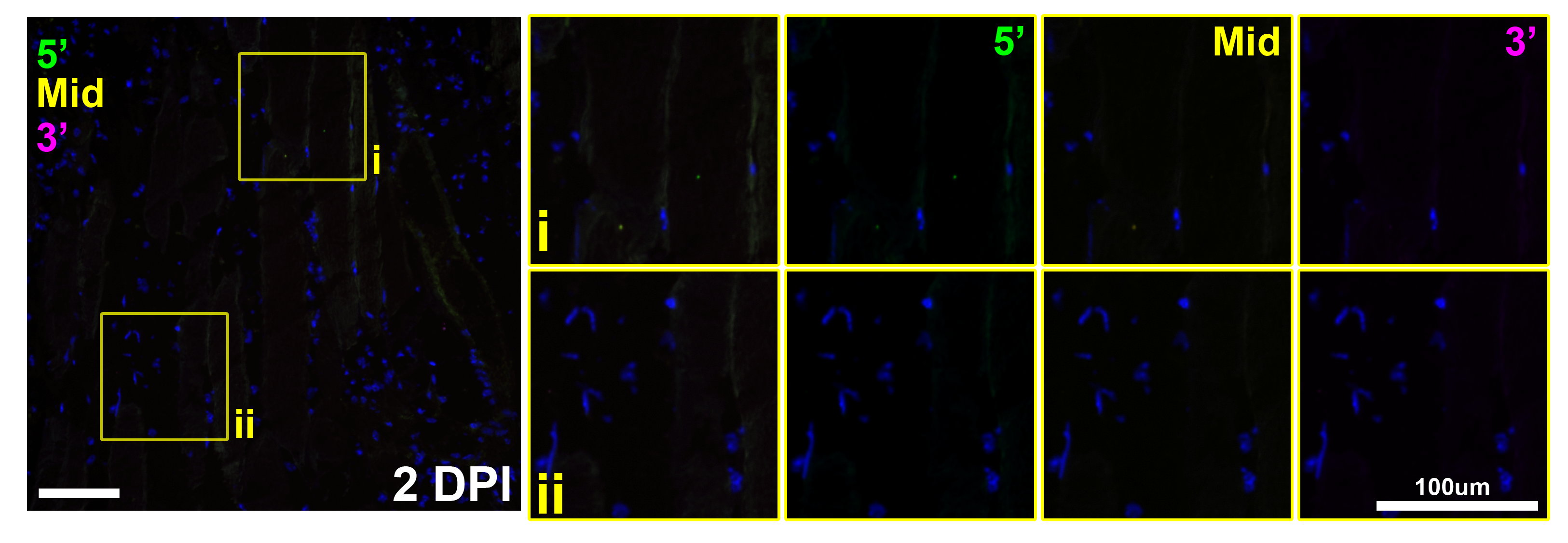

Supplement: Supplementary file 2 — Supplementary Material 2: Supplementary Fig. 2: Dystrophin ISH signal is absent at 2 DPI. Multiplex labelling of dystrophin mRNA (5’: green; middle: yellow; 3’: magenta) in injured muscle at 2 days post injury reveals no labelling with any probe, indicating absence of both mature and nascent transcripts. [file 13395_2025_398_MOESM2_ESM.tif]

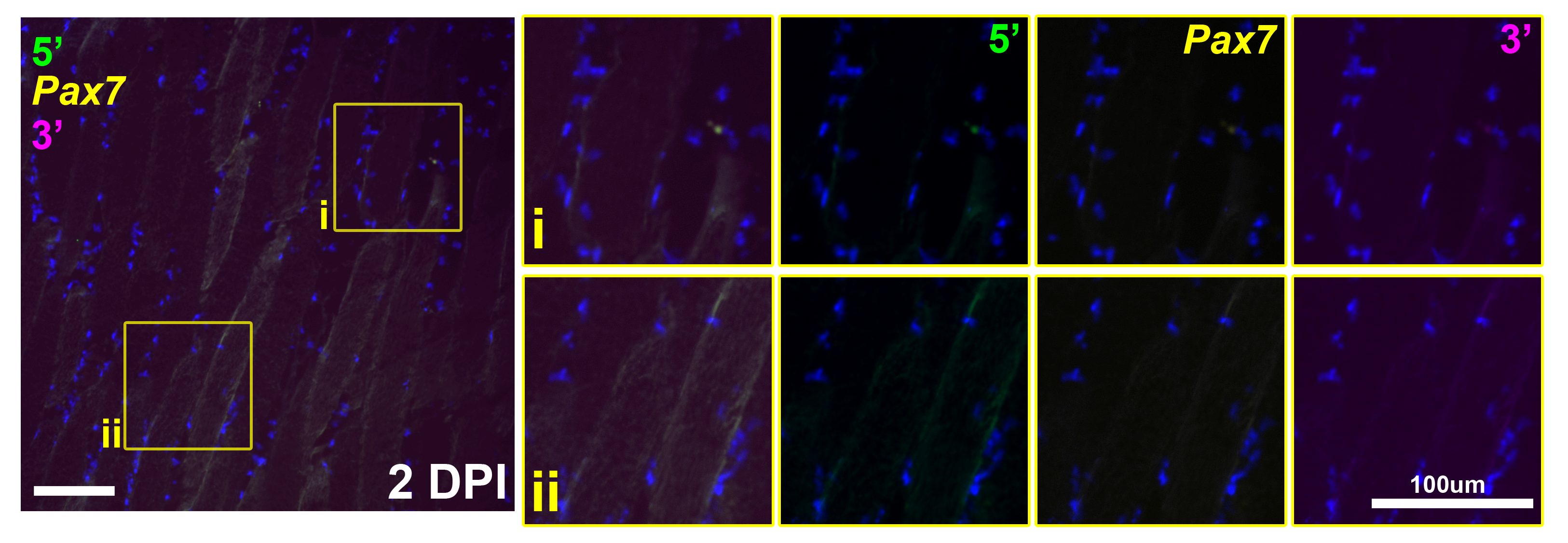

Supplement: Supplementary file 3 — Supplementary Material 3: Supplementary Fig. 3: Pax7 expression is not detected at 2 DPI. Multiplex labelling of dystrophin and Pax7 mRNA (dp427 5’: green; dp427 3’: magenta; pax7: yellow) in injured muscle at 2 days post injury reveals no labelling with any probe, indicating absence of nascent and mature dp427 transcripts, and no detectable pax7 expression. [file 13395_2025_398_MOESM3_ESM.tif]

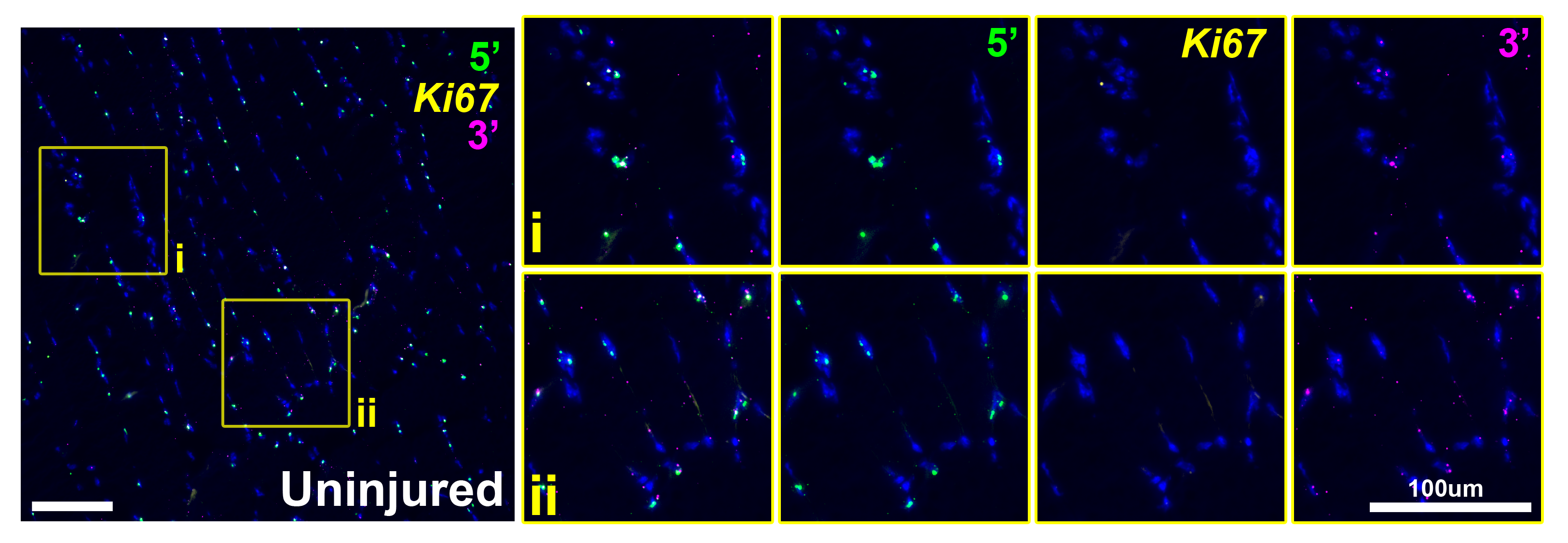

Supplement: Supplementary file 4 — Supplementary Material 4: Supplementary Fig. 4: Ki67 expression is minimal in healthy muscle. Multiplex labelling of dystrophin and Ki67 mRNA (dp427 5’: green; dp427 3’: magenta; ki67: yellow) reveals the characteristic strong nuclear 5’ signals of nascent dp427 mRNAs and the punctate sarcoplasmic 5’ and 3’ signals of mature transcripts, while Ki67 labelling is very low and highly sporadic, found only within rare individual nuclei. [file 13395_2025_398_MOESM4_ESM.tif]
